# Carbon‐Based Composite Phase Change Materials for Thermal Energy Storage, Transfer, and Conversion

**DOI:** 10.1002/advs.202001274

**Published:** 2021-03-03

**Authors:** Xiao Chen, Piao Cheng, Zhaodi Tang, Xiaoliang Xu, Hongyi Gao, Ge Wang

**Affiliations:** ^1^ Institute of Advanced Materials Beijing Normal University Beijing 100875 P. R. China; ^2^ Beijing Advanced Innovation Center for Materials Genome Engineering Beijing Key Laboratory of Function Materials for Molecule & Structure Construction School of Materials Science and Engineering University of Science and Technology Beijing Beijing 100083 P. R. China

**Keywords:** advanced multifunctionality, carbon materials, phase change materials, thermal conductivity, thermal energy storage and conversion

## Abstract

Phase change materials (PCMs) can alleviate concerns over energy to some extent by reversibly storing a tremendous amount of renewable and sustainable thermal energy. However, the low thermal conductivity, low electrical conductivity, and weak photoabsorption of pure PCMs hinder their wider applicability and development. To overcome these deficiencies and improve the utilization efficiency of thermal energy, versatile carbon materials have been increasingly considered as supporting materials to construct shape‐stabilized composite PCMs. Despite some carbon‐based composite PCMs reviews regarding thermal conductivity enhancement, a comprehensive review of carbon‐based composite PCMs does not exist. Herein, a systematic overview of recent carbon‐based composite PCMs for thermal storage, transfer, conversion (solar‐to‐thermal, electro‐to‐thermal and magnetic‐to‐thermal), and advanced multifunctional applications, including novel metal organic framework (MOF)‐derived carbon materials are provided. The current challenges and future opportunities are also highlighted. The authors hope this review can provide in‐depth insights and serve as a useful guide for the targeted design of high‐performance carbon‐based composite PCMs.

## Graphical abstract

This review provides a systematic overview of various carbon‐based composite PCMs for thermal energy storage, transfer, conversion (solar‐to‐thermal, electro‐to‐thermal and magnetic‐to‐thermal), and advanced multifunctional applications, including novel metal organic framework‐derived carbon materials. The current challenges and future opportunities are also highlighted.

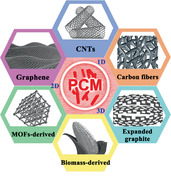

## 1 Introduction

Energy conservation has become an important issue for human activity. Among natural energy sources, thermal energy is conventionally considered a low‐grade type of energy and is generally treated as waste in industrial production. Although solar radiation continuously provides abundant thermal energy during the daytime, a large quantity is often wasted. If abundant thermal energy can be stored and released during supply and demand cycles, the consumption of fossil fuels can be reduced, which will greatly alleviate current energy and environmental issues. Thermal energy storage (TES) techniques are classified into thermochemical energy storage, sensible heat storage, and latent heat storage (LHS).^[^ ^1^, ^2^, ^3^ ^]^ Comparatively, LHS using phase change materials (PCMs) is considered a better option because it can reversibly store and release large quantities of thermal energy from the surrounding environment with small temperature variation during the phase change process.^[^ ^4^, ^5^ ^]^ In contrast to inorganic PCMs, organic PCMs have been extensively studied in the preparation of high‐performance multifunctional composite PCMs.^[^ ^6^, ^7^, ^8^ ^]^ However, although organic PCMs demonstrate great potential for enhancing energy utilization efficiency, their practical applications are hindered by liquid leakage, low thermal conductivity for phonon transmission, weak photoabsorption capacity for solar‐to‐thermal conversion and low electrical conductivity for electric‐to‐thermal conversion.^[^ ^8^, ^9^, ^10^, ^11^ ^]^

Liquid leakage from melted PCMs is potentially dangerous. To date, the most popular leak‐proof strategies are the construction of shape‐stabilized composite PCMs and core–shell composite PCMs.^[^ ^12^, ^13^, ^14^, ^15^, ^16^, ^17^, ^18^, ^19^, ^20^ ^]^ Low thermal conductivity decelerates the thermal charging/discharging rates of PCMs. Originally, additives with high thermal conductivity were directly introduced into a system to obtain thermally conductive composite PCMs; however, the enhancement of thermal conductivity was generally limited. Moreover, this strategy inevitably reduces the TES density due to the occupation of the inherent pores.^[^ ^4^, ^15^, ^21^, ^22^, ^23^, ^24^, ^25^, ^26^, ^27^ ^]^ To balance these two contradictions, 3D interconnected carbon materials have been considered as supports to encapsulate PCMs, which are competitive candidates for the preparation of composite PCMs with high thermal conductivity.^[^ ^24^, ^27^, ^28^, ^29^ ^]^ Additionally, to address the weak photoabsorption and low electrical conductivity of pristine PCMs, introducing carbonaceous materials into a system is one prospective method due to their excellent photoabsorption and high electrical conductivity. Relevant reports have also shown that introducing small amounts of carbon materials into pristine PCMs can effectively improve the photoabsorption and electrical conductivity of PCMs by constructing a carbon percolation network. Therefore, carbon‐based composite PCMs have become increasingly attractive in solar‐to‐thermal, electric‐to‐thermal, and even magnetic‐to‐thermal conversions.^[^ ^8^, ^11^, ^29^, ^30^, ^31^, ^32^, ^33^, ^34^ ^]^

**Figure 1:**
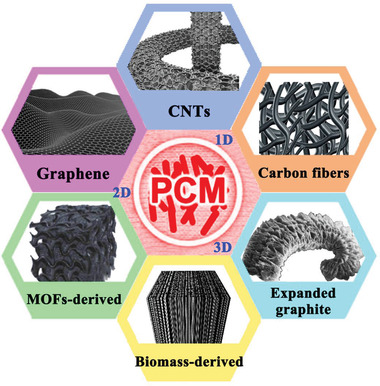
Overview of various carbon materials for TES, transfer, and conversion.

Very recently, numerous high‐performance carbon‐based composite PCMs have been reported. Although there are some published reviews on composite PCMs,^[^ ^4^, ^35^, ^36^, ^37^, ^38^, ^39^, ^40^, ^41^, ^42^, ^43^, ^44^ ^]^ they have mainly focused on thermal storage and thermal transfer enhancement, and the corresponding thermal enhancement mechanisms have mainly focused on structural level analysis and have lacked microscale phonon mechanism analysis.^[^ ^4^, ^26^, ^43^ ^]^ An informative review providing constructive microscale phonon insights into advanced carbon‐based composite PCMs for thermal storage, thermal transfer, energy conversion, and advanced utilization does not exist. Herein, we summarize the recent advances in high‐performance carbon‐based composite PCMs for thermal storage, thermal transfer, energy conversion, and advanced utilization, which mainly include carbon nanotubes (CNTs), carbon fibers (CFs), graphene/GO/rGO, metal organic frameworks (MOFs)‐derived carbon, biomass‐derived carbon, expanded graphite (EG), and other carbon materials (**Figure** **1**). In particular, we analyze the thermal transfer micromechanisms from the perspective of lattice vibration and phonon transmission. Finally, the perspectives and challenges in the development of high‐performance carbon‐based composite PCMs are highlighted.

## 2 Classification and Confinement Strategy of PCMs

### 2.1 Classification of PCMs

**Figure 2:**
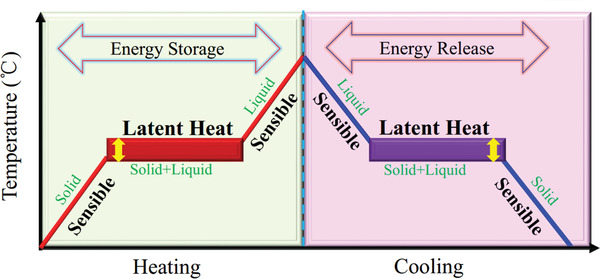
The working principle of solid–liquid PCMs. Reproduced with permission.^[^ ^52^ ^]^ Copyright 2020, Elsevier.

PCMs are functional materials that can reversibly absorb and release large amounts of latent heat during the phase change processes.^[^ ^45^, ^46^, ^47^ ^]^ Usually, PCMs can be classified according to the phase change states, melting temperature ranges, or chemical compositions.^[^ ^3^, ^48^, ^49^ ^]^ According to the phase change states, PCMs can be divided into four categories: solid–solid PCMs, solid–liquid PCMs, liquid–gas PCMs, and solid–gas PCMs. Comparatively, liquid–gas and solid–gas PCMs exhibit the highest amount of latent heat storage; however, large volumetric shrinkage during the phase change process and specialized pressurized containers hinder their practical application. Solid–solid PCMs usually exhibit low TES density and high phase change temperature. Solid–liquid PCMs exhibit suitable phase change temperature and high latent heat. Therefore, solid‐liquid PCMs are the most practical candidates and are also the most widely studied PCMs in recent years.^[^ ^35^, ^50^, ^51^ ^]^ The working principle of solid‐liquid PCMs is shown in **Figure** **2**.

**Figure 3:**
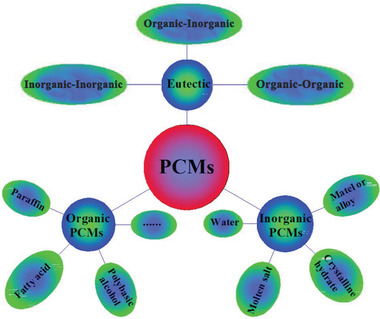
Classification of PCMs based on chemical compositions. Reproduced with permission.^[^ ^40^ ^]^ Copyright 2020, Elsevier.

According to the melting temperature (*T*_m_) range, PCMs can be divided into three categories: low temperature PCMs (*T*_m_<100 °C), medium temperature PCMs (100 °C<*T*_m_<250 °C) and high temperature PCMs (*T*_m_>250 °C).^[^ ^53^ ^]^ According to the chemical compositions, PCMs can be divided into three categories: organic PCMs, inorganic PCMs, and eutectic PCMs (**Figure** **3**). Eutectic PCMs are a mixture of organic PCMs and inorganic PCMs. Inorganic PCMs include water, salts, salt hydrates, and metals, which exhibit high volumetric TES density and relatively high thermal conductivity (0.40–0.70 W mK^−1^). However, severe phase separation and super cooling phenomena strongly hinder their large‐scale application. Organic PCMs include paraffin waxes, fatty acids, and polymers. Comparatively, organic PCMs exhibit relatively high phase change enthalpy and reasonable phase change temperature, and they have no phase separation and weak super cooling phenomena. Organic PCMs are also easy to handle, abundantly available, and relatively inexpensive. Therefore, organic PCMs are the most popular in the practical applications. However, their low thermal conductivity (0.15–0.30 W mK^−1^) will result in inadequate heat transfer that prevents heat from penetrating into the PCM interior, thereby reducing their effectiveness in TES applications. In addition, organic PCMs also have leakage issue and flammability issue. Comparative advantages and disadvantages of organic PCMs and inorganic PCMs are listed in **Table** **1**. In this review, we mainly summarize organic PCMs confined in carbon materials for thermal storage, thermal transfer, energy conversion, and advanced utilization.

**Table 1:** Advantages and disadvantages of organic and inorganic PCMs. Reproduced with permission.^[^ ^48^ ^]^ Copyright 2014, Wiley‐VCH. Reproduced with permission.^[^ ^54^ ^]^ Copyright 2009, Elsevier

Classification		Advantages	Disadvantages
Organic PCMs	Paraffin	Availability in a broad range of temperatures	Low thermal conductivity
		Chemically stable and inert	Moderately flammable
		High heat of fusion	Relatively high costs of pure paraffins
		No or minor supercooling	Noncompatible with plastic containers
		Noncorrosive	–
	Non‐paraffin	High heat of fusion	Low thermal conductivity
		No or minor supercooling (depending on form of application)	Flammable
			Instability at high temperatures
Inorganic PCMs	Salt hydrate	Sharp melting point	Phase separation
		High thermal conductivity	Cycling stability
		High heat of fusion per unit	Supercooling
		Small volume change	Corrosive
		Low cost	
	Metal	Sharp melting points	Corrosive
		High thermal conductivity	Toxicity, environmental concerns
		Small volume change	

### 2.2 Confinement Strategy of PCMs

Confinement is the sealing process of PCMs to prepare shape‐stabilized composite PCMs using supporting materials.^[^ ^2^, ^48^, ^55^ ^]^ The confinement strategy aims to solve the leakage issue of melted PCMs and stop the problematic contact between PCMs and the interactive environment.^[^ ^48^ ^]^ Importantly, the surface chemistry involved at the interface between supporting materials and PCMs significantly affects the thermodynamic performances of PCMs. Therefore, confinement strategies can effectively adjust the thermophysical properties of PCMs. Usually, confinement strategies can be classified according to the pore size and dimensions of the supporting materials.^[^ ^46^ ^]^ Confinement strategies can be classified into nanoconfinement, microconfinement, and macroconfinement according to the pore sizes of the supporting materials.

**Figure 4:**
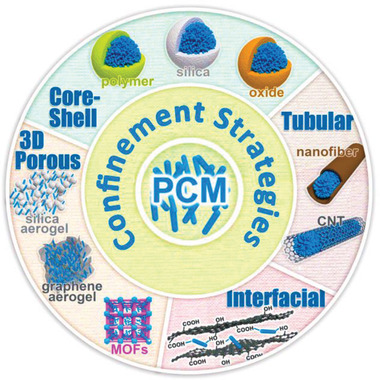
Different dimensional confinement strategies of PCMs. Reproduced with permission.^[^ ^46^ ^]^ Copyright 2018, Royal Society of Chemistry.

Recent studies have shown that nanoconfinement strategy is more efficient than other confinement strategies due to its large specific surface area, abundant surface functionality, and high thermal transfer ability.^[^ ^46^, ^55^ ^]^ According to the dimensions of the supporting materials, nanoconfinement strategies can be further classified into core–shell confinement (0D), longitudinal confinement (1D), interfacial confinement (2D), and porous confinement (3D), as shown in **Figure** **4**.^[^ ^46^ ^]^ In core–shell confinement, the shell can ensure the structural integrity and stability of composite PCMs. In longitudinal confinement, a highly aligned structure can improve the thermal storage performance of composite PCMs. In interfacial confinement, strong interface‐induced interactions can effectively hinder the fluidity of melted PCMs. In porous confinement, strong pore‐induced capillary action can stabilize PCMs, thereby providing shape stability. The pore size determines the confinement efficiency of PCMs. Pores can be classified into micropores (<2 nm), mesopores (2–50 nm), and macropores (>50 nm) based on the pore diameter.^[^ ^56^ ^]^ Typically, smaller micropores may restrict the phase change behaviors of PCMs, whereas larger macropores are not sufficiently adequate to stabilize the melted PCMs. Comparatively, mesopores and smaller macropores are more suitable in the preparation of shape‐stabilized composite PCMs.

## 3 Thermal Transfer Mechanism of PCMs

### 3.1 Lattice Vibration and Phonon Transmission

Currently, although many studies have been devoted to improving the thermal conductivity of PCMs, there are still relatively few reports on thermal conductivity enhancement from the perspective of micromechanisms. The electrons and lattice vibrations in solids enable thermal transfer.^[^ ^57^ ^]^ In detail, thermal transfer is dominated by electrons in metallic PCMs, whereas thermal conduction is dominated by lattice vibrations in nonmetallic PCMs.^[^ ^40^ ^]^ Although some nonmetallic PCMs have electrons bound in their ionic crystal lattice, free electrons are rarely sufficient for thermal conduction. For nonmetallic PCMs, the thermal transfer mechanism is mainly lattice vibration. Phonons come from lattice vibrations and are the quanta of lattice vibrations. Therefore, phonons are the dominant thermal carriers in nonmetallic PCMs at the microscopic level.

**Figure 5:**
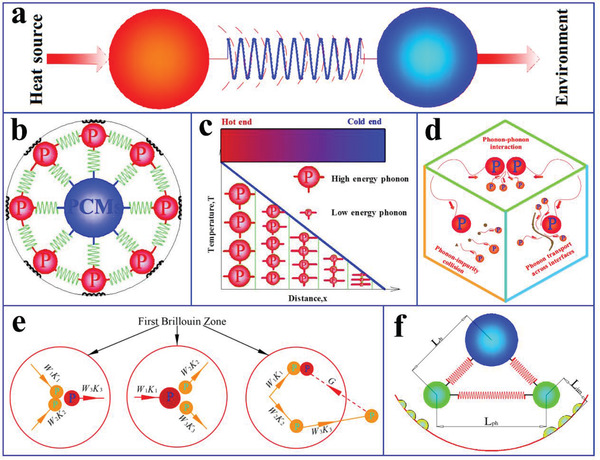
a) Heat transfer scheme in crystal heat conduction. b) Spring vibration heat transfer system. c) Temperature curve with length and phonon energy diagram. d) Phonon scattering modes. e) Phonon–phonon collision modes: N and U processes. f) Phonon mean free pathways for three different phonon scattering modes. Reproduced with permission.^[^ ^40^ ^]^ Copyright 2020, Elsevier.

Phonons, as the driving force responsible for heat transfer, are the most basic factor for describing thermal conduction. The three main factors affecting phonon transmission are phonon velocity, specific heat capacity, and mean free path. During phonon transmission, phonon diffusion and phonon scattering are two opposing factors in terms of heat conduction. The stronger the phonon diffusion is, the higher the thermal conductivity is. Conversely, the stronger the phonon scattering is, the lower the thermal conductivity is. Phonon scattering can change or even reverse the direction of energy transfer, thereby becoming the dominant reason for deterioration in phonon diffusion. Theoretically, if phonons can be randomly transmitted without any hindrance, it is possible to obtain an ideal thermal conductivity. In nonmetallic composite PCMs, phonon scattering caused by the mismatch between supporting materials and PCMs induces large interfacial thermal resistances and reduces the thermal conductivity. The abovementioned carbon materials with an interconnected 3D network can provide efficient thermal transfer pathways and continuous channels for phonon propagation. Moreover, close contact between PCMs and supporting materials can reduce the interfacial thermal resistance. Phonon scattering can be divided into the following three categories: phonon–phonon scattering, phonon–defect scattering, and phonon–interface scattering.^[^ ^58^ ^]^ The individual parameters of the phonon scattering mechanism on the effect of phonon heat conduction are shown from a microscopic point of view in **Figure** **5**.

### 3.2 Phonon Scattering

#### 3.2.1 Phonon–Phonon Scattering

Phonons have the pronounced particle‐like vibrational pattern of a crystal; therefore, phonons can be considered quasiparticles. Many phonons are involved in the interactions occurring during the wave propagation process. Nevertheless, the number of phonons is not conserved because of generation or annihilation. Three‐phonon scattering is the most significant interaction among waves and determines the thermal conductivity. Three‐phonon scattering exhibits two patterns: 1) two phonons interact and combine into a third phonon; and 2) the energy mode is divided from one to two. Usually, phonon–phonon collisions are classified into normal processes (N processes) and Umklapp processes (U processes).^[^ ^59^ ^]^ The N processes follow both energy conservation and momentum conservation, whereas the U processes follow only energy conservation. If phonons have no interactions, thermal energy will propagate across the perfect crystal, and the thermal conductivity will be infinite. In fact, thermal energy does not simply proceed straight from one end to the other, but undergoes frequent collisions through the crystals. The average distance between collisions is called the mean free path. In theory, analyzing the mean free path of phonons is very complicated. Herein, the temperature is simply divided into three stages: low temperature, medium temperature, and high temperature. At a sufficiently low temperature, lattice vibrations will be frozen, and phonons will be difficult to be excited. As a result, the chance of collisions among the few excited phonons is very small, thereby extending the mean free path. Under these conditions, phonon‐boundary scattering is the dominant mechanism. The thermal conductivity is proportional to *T* ^3^ (*T* is the absolute temperature). With increasing temperature, the chance of collisions among excited phonons also increases. Consequently, the mean free path becomes shorter. At high temperature, although the number of excited phonons is proportional to *T*, the mean free path is proportional to 1/*T*. The anharmonic coupling effect between different phonons restricts the mean free path. Hence, the thermal conductivity is proportional to 1/*T*.^[^ ^40^ ^]^

#### 3.2.2 Phonon‐Boundary Scattering

Phonon‐boundary scattering is the dominant mechanism at low temperatures. When phonons travel through the crystal boundary, the resulting scattering phenomenon affects the mean free path of the phonons, causing the longest mean free path.^[^ ^40^ ^]^ When phonons are close to the boundary, they produce two types of boundary scattering: specular phonon‐boundary scattering and diffuse phonon‐boundary scattering. In specular phonon‐boundary scattering, the reflection of the phonon is specular. In diffuse phonon‐boundary scattering, phonons are scattered at various angles. Both of these phonon‐boundary scattering modes have an adverse effect on thermal transfer, causing interfacial thermal resistances due to the acoustic mismatch across the interfaces.^[^ ^60^ ^]^

#### 3.2.3 Phonon‐Defect Scattering

Some defects inevitably occur in the crystal lattice, and these defects mainly include point defects, dislocations, vacancy defects, and impurities. These lattice defects are detrimental to heat transfer because they can cause phonon scattering, such as phonon reflection, diffraction, or refraction.^[^ ^40^ ^]^ Usually, a small number of impurities exist in the crystal structure; however, they can have a significant influence on the thermal conductivity. These impurities inevitably generate additional thermal resistance, thereby resulting in a decrease in thermal conductivity. In short, existing lattice defects will shorten the mean free path, resulting in a lower thermal conductivity. Therefore, phonon‐defect scattering is detrimental to phonon transmission.

In summary, the internal heat conduction in materials is the result of the collision of various internal microparticles. Therefore, a change in crystal microstructure will directly influence thermal conductivity. The three factors directly affecting the thermal conductivity of composite PCMs are phonon group velocity, specific heat capacity, and phonon mean free path. The abovementioned three kinds of phonon scattering mechanisms (phonon–phonon scattering, phonon–boundary scattering, and phonon–defect scattering) inevitably produce thermal resistance in composite PCMs, thus reducing their thermal conductivity. In terms of the thermal transfer mechanism of PCMs and carbon materials, the interface configurations of the two components are particularly important for thermal transfer. After carbon materials are introduced into PCMs, numerous sensitive thermal interfaces are usually formed due to the high specific surface area and porosity of carbon materials, and interfacial thermal resistance is inevitable. Lattice vibration and phonon transmission facilitate thermal transfer while phonon scattering hinders thermal transfer. 1D carbon materials can establish linear thermal transfer paths, 2D carbon materials can establish planar thermal transfer paths, and 3D carbon materials can establish networked thermal transfer paths. Theoretically, the thermal transfer efficiency of different dimensional carbon materials follows the tendency of 3D > 2D > 1D. Generally, continuous interpenetration of 3D network carbon materials can further accelerate lattice vibration and phonon transmission, and reduce phonon scattering by constructing low interface thermal resistances, thus improving the thermal conductivity of carbon‐based composite PCMs. In addition, surface functionalization and doping strategies can also effectively accelerate lattice vibration and phonon transmission and reduce phonon scattering.

## 4 Overview of Carbon Materials

**Figure 6:**
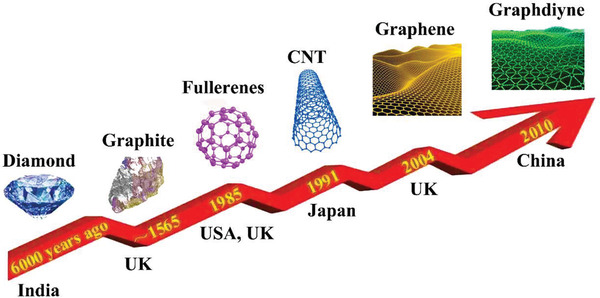
The development of carbon family.

**Figure 7:**
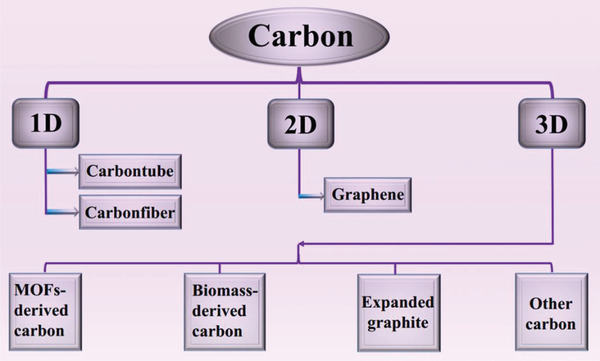
Summary of various carbon materials in this review.

Carbon‐based materials have attracted widespread interest in many disciplines due to several factors, including their unique structure and their thermal and electrical characteristics.^[^ ^4^, ^61^, ^62^, ^63^ ^]^ To date, carbon materials have sequentially developed from diamond, graphite, fullerenes, CNTs, graphene to graphdiyne (**Figure** **6**). According to the dimensions, carbon materials are classified into 0D (carbon dots), 1D (carbon tubes/fibers), 2D (graphene), and 3D (porous carbon network) in this review. Considering the inherent fluorescence characteristics, carbon dots can be used to develop fluorescent functional composite PCMs. In terms of the leakage issue and low thermal conductivity of pure organic PCMs, 1D, 2D, and 3D carbon‐based porous materials (**Figure** **7**) have attracted more popularity for confining organic PCMs due to their unique features, such as high exposed surface area, adjustable functional surface, high‐temperature stability, high intrinsic thermal conductivity, and noncorrosive nature.

In brief, CNTs and CFs have a conspicuous 1D cylindrical structure with a high length‐to‐diameter ratio. As containers of organic PCMs, CNTs or CFs should have large internal diameters, short lengths, open ends, and excellent wettability.^[^ ^64^ ^]^ Long capped CNTs or CFs are commonly cut into shorter open‐ended pieces. The chemical functionalization of CNTs or CFs is usually modified to introduce oxygen‐rich functional groups via different types and concentrations of acid treatment, thereby illustrating great potential for the preparation of composite PCMs.^[^ ^65^ ^]^ More attractively, in the longitudinal direction, single‐walled carbon nanotubes (SWCNTs) have an extremely high thermal conductivity of ≈3500 W mK^−1^ and multiwalled carbon nanotubes (MWCNTs) have a thermal conductivity of ≈3000 W mK^−1^.^[^ ^66^ ^]^ Graphene is originally mechanically exfoliated from graphite. Graphene and SWCNTs are both single‐layer carbon atoms with the same layer thickness of 0.34 nm and the same surface area per mass of ≈2630 m^2^ g^−1^. More importantly, as a container of PCMs, graphene has an extremely high thermal conductivity of ≈5000 W mK^−1^, which is even higher than that of SWCNTs.^[^ ^4^, ^67^ ^]^ Graphene oxide (GO) is the most typical derivative of graphene. Graphene could also be obtained from GO through thermal reduction or chemical reduction. Compared with the direct synthesis of graphene, GO reduction method is easier and low‐cost.

Recently, 3D porous carbon materials have been widely utilized for thermal performance enhancement of composite PCMs. Compared to 1D and 2D carbon materials, 3D carbon‐based materials have more structural advantages, including higher porosity, higher specific surface area, larger thermal storage capacity, higher thermal conductivity, and 3D shape stability.

## 5 Carbon‐Based Composite PCMs for Thermal Energy Storage, Transfer, and Conversion

Carbon materials are the most popular additives for the thermal performance enhancement of composite PCMs. To provide systematic insights and guidance for the preparation of high‐performance carbon‐based composite PCMs, we mainly summarize CNTs, carbon fibers (CFs), graphene/GO/rGO, MOF‐derived carbon, biomass‐derived carbon, expanded graphite (EG), and other porous carbons (PCs) in this review (**Figure** **7**). The advantages and disadvantages of various carbon materials for TES, transfer, and conversion are shown in **Table** **2**. In addition to exhibiting excellent thermal storage and transfer performances (**Table** **3**), some carbon‐based composite PCMs exhibit attractive multifunctionality, such as solar‐to‐thermal conversion, electric‐to‐thermal conversion, magnetic‐to‐thermal conversion, thermotherapy, and fluorescence functionalities.

**Table 2:** Advantages and disadvantages of various carbon materials for TES, transfer and conversion

Carbon materials	Advantages	Disadvantages
CNTs	High loading	Complex preparation process
	High conductivity	Easy agglomeration
	Strong solar absorption capacity	High cost
	High conversion efficiency	
Carbon fibers	High loading	Easy agglomeration
	High conductivity	
	Low cost	
Graphene/GO/rGO	High specific surface area	Complex preparation process
	High loading	Easy agglomeration
	High conductivity	High cost
	Strong solar absorption capacity	
	High conversion efficiency	
MOF‐derived carbon	High porosity	Easy to collapse at high temperature
	High specific surface area	Complex preparation process
	Adjustable pore structure	Low thermal conductivity
	High loading	High cost
	High conversion efficiency	
Biomass‐derived carbon	Wide sources of raw materials	Relatively low conversion efficiency
	Green and pollution‐free	Low thermal conductivity
	Low cost	
Expanded graphite	Large pore volume	Large expansion coefficient
	Low density	
	High loading	
	High conductivity	
	Low cost	

**Table 3:** Summary of carbon‐based organic PCMs for TES

Supporting materials	Type of PCMs	Loading [wt%]	Melting point [°C]	Freezing point [°C]	Melting enthalpy [J g^−1^]	Freezing enthalpy [J g^−1^]	Thermal conductivity [W mK^−1^]	Ref.
CNTs	PEG	96.0	61.1	38.9	92.6	92.7	–	^[^ ^73^ ^]^
CNTs array	*n*‐eicosane	90.0	34.5	–	241.0	–	–	^[^ ^78^ ^]^
CNTs sponge	Paraffin	91.0	27.0	–	138.2	–	–	^[^ ^77^ ^]^
CNTs sponge	Sebacic acid	60.0	121.1	120.7	131.8	130.2	7.27	^[^ ^75^ ^]^
CNTs sponge/polyurethane	Polyurethane	80.0	60.3	42.2	119.4	113.9	2.40	^[^ ^25^ ^]^
CNTs‐Cu foam	Paraffin	–	59.9	55.0	–	–	3.49	^[^ ^26^ ^]^
CNTs/CFs/epoxy	Paraffin	19.5	45.1	33.9	36.4	36.2	–	^[^ ^88^ ^]^
Carbon fibers (CFs)	Paraffin	91.2	57.0	48.8	199.4	199.2	0.77	^[^ ^15^ ^]^
Ethylene‐vinyl acetate/EG‐CFs	Paraffin	–	49.0	–	169.7	–	–	^[^ ^87^ ^]^
Graphene	Paraffin	97.0	60.8	49.1	200.0	197.0	0.62	^[^ ^109^ ^]^
Graphene	Paraffin	–	57.1	47.8	161.0	157.9	0.92	^[^ ^114^ ^]^
Graphene	PEG	–	56.4	43.7	169.3	157.6	3.11	^[^ ^32^ ^]^
GO	PEG	91.0	65.3	42.6	158.2	157.5	0.48	^[^ ^99^ ^]^
Graphene/Ag	PEG	92.0	60.3	36.1	166.1	167.8	0.41	^[^ ^106^ ^]^
Graphene/cellulose	PEG	97.0	61.1	37.0	182.6	177.7	1.03	^[^ ^100^ ^]^
GO/BN	PEG	79.8	65.5	42.1	157.7	150.6	1.72	^[^ ^113^ ^]^
GO/BN	PEG	81.4	65.7	42.2	145.9	139.3	1.84	^[^ ^115^ ^]^
GO/BN	PEG	72.6	64.8	42.6	143.6	136.0	1.89	^[^ ^113^ ^]^
GO/graphene/melamine foam	Paraffin	–	51.6	48.8	154.1	161.7	1.46	^[^ ^98^ ^]^
EG	Stearic acid	94.0	53.5	53.6	163.5	167.3	2.50	^[^ ^179^ ^]^
EG	MeSA	–	33.5	34.4	145.0	146.0	3.60	^[^ ^190^ ^]^
EG/SiO_2_	Hexadecane	73.3	20.1	16.1	147.6	145.1	–	^[^ ^188^ ^]^
EG/HDPE	Paraffin	60.0	56.3	37.3	90.9	–	–	^[^ ^176^ ^]^
EG/PVB	Palmitic acid	70.0	59.5	56.4	128.1	132.9	0.51	^[^ ^175^ ^]^
EG/silicone elastomer	Octadecanol	–	55.7	–	234.1	–	1.53	.^[^ ^187^ ^]^
EG/Co_3_O_4_	Stearic acid	–	69.4	68.3	192.5	192.8	2.53	^[^ ^53^ ^]^
EG/OBC	Palmitic acid	79.2	50.7	–	197.7	–	5.50	^[^ ^184^ ^]^
EG/CaCO_3_	Paraffin	–	48.0	–	115.2	–	7.20	^[^ ^174^ ^]^
EG/CaCO3	Paraffin	–	49.3	–	96.8	–	10.37	^[^ ^173^ ^]^
Winter melon carbon	Paraffin	95.0	53.5	48.3	115.2	126.9	–	^[^ ^158^ ^]^
Wood carbon	Tetradecanol	–	36.9	37.0	119.2	104.3	0.22	^[^ ^172^ ^]^
Wood carbon	Lauric acid	81.1	41.0	38.9	177.9	178.2	0.27	^[^ ^157^ ^]^
Adromischus cooperi carbon	Paraffin	95.0	61.9	56.5	133.1	147.7	0.43	^[^ ^159^ ^]^
Corn cob carbon	Lauric‐stearic acid	77.9	35.1	29.7	148.3	144.2	0.44	^[^ ^166^ ^]^
Cotton carbon	Hexadecanol	98.5	50.8	–	219.4	–	0.40	^[^ ^137^ ^]^
Cotton carbon	Paraffin	95.5	57.7	49.6	209.3	207.9	0.43	^[^ ^171^ ^]^
Fungi carbon	Stearic acid	77.5	52.7	53.0	144.8	142.6	0.86	^[^ ^168^ ^]^
Potato carbon	PEG	85.4	56.5	37.9	175.6	158.5	4.50	^[^ ^161^ ^]^

### 5.1 CNT‐Based Composite PCMs

Theoretically, the thermal conductivity of SWCNTs and MWCNTs can reach 3000 W mK^−1^ in the longitudinal direction.^[^ ^66^ ^]^ This superior thermal performance of CNTs shows endless potential for TES and transfer materials. In addition to exhibiting high thermal conductivity, CNTs have been thoroughly investigated in the phase change TES field due to desirable properties, such as low density, high surface area, large pore volume, high photoabsorption coefficient, and excellent electrical conductivity.

Some impressive results have been obtained regarding CNT‐based composite PCMs. It is worth noting that the distribution of CNTs in PCMs has an important influence on the thermal conductivity of CNT‐based composite PCMs. Comparatively, an arrayed CNTs distribution usually provides more advantages than a random CNTs distribution in terms of the thermal conductivity enhancement of PCMs. For example, Sarı et al.^[^ ^68^ ^]^ introduced random CNTs into expanded vermiculite clay/capric acid‐stearic acid (SA) eutectic mixture composite PCMs for the thermal conductivity enhancement. However, the thermal conductivity of composite PCMs was only 0.43 W mK^−1^. Similarly, Tang et al.^[^ ^69^ ^]^ introduced random CNTs into polyethylene glycol (PEG)/SiO_2_ composite PCMs. The resulting PEG/SiO_2_/CNT composite PCMs showed an increased thermal conductivity of 0.46 W mK^−1^. Tao et al.^[^ ^70^ ^]^ prepared two types of random CNT‐based composite PCMs with Li_2_CO_3_‐K_2_CO_3_ binary carbonate eutectic salts as the PCMs. Compared with MWCNT‐based composite PCMs, SWCNT‐based composite PCMs are more conducive to obtaining higher thermal conductivity.

**Figure 8:**
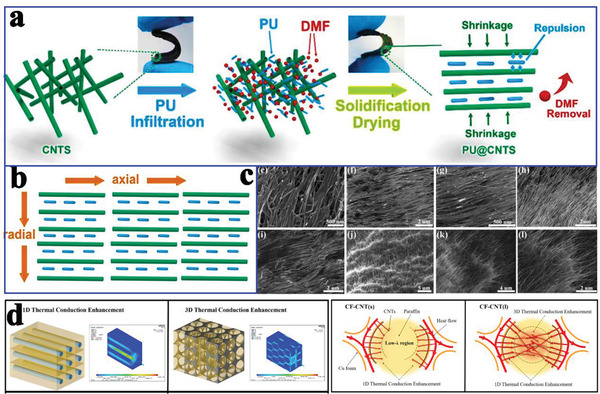
a) Preparation diagram of PU@CNTs. b) Schematic illustration of thermal conduction in PU@CNTs along the axial and radial directions. c) SEM images of PU@CNTs‐10 from top surface, cross‐section, bottom surface, center, and edge. Reproduced with permission.^[^ ^71^ ^]^ Copyright 2019, Elsevier. d) Simulated heat flux field distributions in paraffin‐based composite PCMs and schematic diagram for thermal conduction inside the pores in CF‐CNTs. Reproduced with permission.^[^ ^26^ ^]^ Copyright 2019, Elsevier.

However, the improvement in thermal conductivity was not as high as expected due to random CNTs inducing greater hindrance of interface thermal conductance in composite PCMs. Moreover, random CNTs will partially agglomerate, which will increase the thermal resistance of the interface. To further reduce the thermal resistance of the interface of random CNT‐based composite PCMs, Aftab et al.^[^ ^71^ ^]^ constructed arrayed CNTs to encapsulate polyurethane (PU) (**Figure** **8**a). The densified and arrayed CNTs in the horizontal direction created a synergistic enhancement in the thermal conductivity (Figure 8b). Along the axial direction, the thermal conductivity of PU@CNTs composite PCMs was 2.40 W mK^−1^, which was ≈10 times higher than that of pure PU. Such excellent thermal conductivity was originated from the arrayed CNTs (Figure 8c).

In addition to the individual use of CNTs as a supporting material for the thermal conductivity enhancement of PCMs, Zhu et al.^[^ ^26^ ^]^ fabricated CNTs‐Cu foam hybrids for such enhancement. In contrast to conventional carbon‐metal hybrids, these CNTs were radially grown on the surface of Cu foam under nickel catalysis. The resulting CNTs‐Cu foam hybrids not only reduced the low‐*λ* regions but also connected every branch, thereby strengthening the integrity of the whole reinforcement. Importantly, the thermal conductivity of composite PCMs was increased to 3.49 W mK^−1^ compared with that of paraffin (0.11 W mK^−1^). This enhancement was attributed to the CNT‐constructed 3D thermal conduction network, which not only expanded the heat transfer area but also improved the thermal conduction inside the pores (Figure 8d).

**Figure 9:**
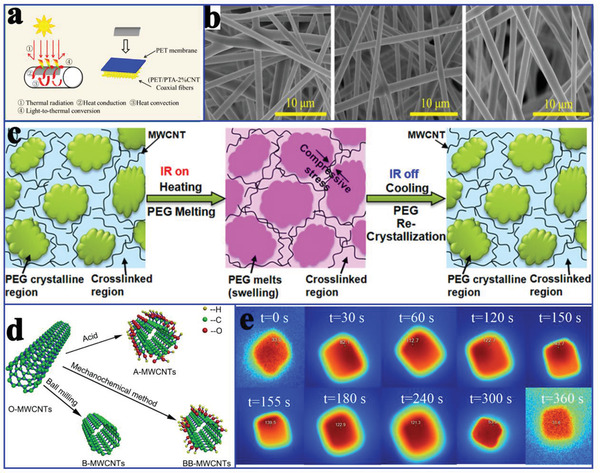
a) Schematic process for the solar‐to‐thermal conversion. b) SEM images of PET/PTA‐*x* CNTs coaxial fibers. Reproduced with permission.^[^ ^72^ ^]^ Copyright 2019, American Chemical Society. c) Conceptual illustration of the cyclic, dramatic, and reversible electrical conductivity changes. Reproduced with permission.^[^ ^73^ ^]^ Copyright 2015, American Chemical Society. d) The ball milling and chemical treatment processes. Reproduced with permission.^[^ ^74^ ^]^ Copyright 2018, Elsevier. e) Infrared images of SA/CNTs sponge under the solar irradiation. Reproduced with permission.^[^ ^75^ ^]^ Copyright 2018, Elsevier.

In addition to popular thermal conductivity enhancement studies, Li et al.^[^ ^72^ ^]^ fabricated comb‐like polymeric PCMs composed of a poly(ethylene terephthalate) (PET) sheath and poly(tetradecyl acrylate) (PTA) core by dispersing CNTs into PET and PTA polymeric substances using electrospinning technology for solar‐to‐thermal conversion (**Figure** **9**a). Scanning electron microscopy (SEM) images indicated that the incorporated CNTs had no influence on the formation of the coaxial fibers (Figure 9b). More importantly, the incorporated CNTs play a crucial role in the solar absorption efficiency. The experimental results indicated that the PET/PTA‐2% CNTs could reach 60 °C after 600 s of illumination (100 mW cm^−2^). Furthermore, the CNT network effectively guaranteed the shape stability during the solar‐to‐thermal conversion process.

Regarding infrared radiation (IR)‐regulated responses and short response times in composite PCMs, Wang et al.^[^ ^73^ ^]^ reported MWCNT‐based composite PCMs, which exhibited IR‐regulated on/off electrical conductivity ratios of 11.6 ± 0.6 and 570.0 ± 70.5 times at IR powers of 7.3 and 23.6 mW mm^−2^, respectively (Figure 9c). The MWCNTs could be modified using three different methods (acid oxidation, mechanochemical process, and ball milling), thereby obtaining three kinds of MWCNTs (A‐MWCNTs, BB‐MWCNTs, and B‐MWCNTs) (Figure 9d).^[^ ^74^ ^]^ Although the acid oxidation process introduced —COOH groups, it also introduced the vacancies into the structure, which caused a sharp reduction in the thermal conductivity of the MWCNTs. Although ball milling treatment shortened the length of the MWCNTs, it also increased the number of random contacts between CNTs and the interfacial thermal resistance. The mechanochemical process helps generate strong chemical bonds on the surface of the MWCNTs, thereby reducing the interfacial thermal resistance. Therefore, the thermal conductivity of erythritol/B‐MWCNTs was lower than that of erythritol/O‐MWCNTs. The thermal conductivity values of erythritol/BB‐MWCNTs and erythritol/A‐MWCNTs were higher than those of erythritol/B‐MWCNTs.

**Figure 10:**
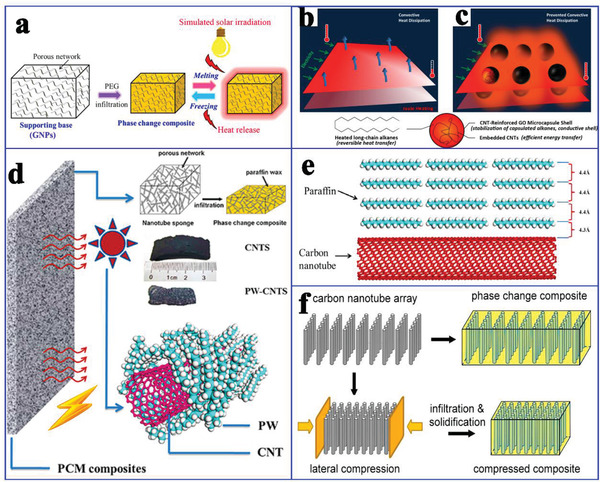
a) Formation mechanism of PEG/GNP. Reproduced with permission.^[^ ^32^ ^]^ Copyright 2018, American Chemical Society. b,c) The role and formation process of C22@GO‐CNTs microcapsules. Reproduced with permission.^[^ ^76^ ^]^ Copyright 2016, American Chemical Society. d) Scheme of electric‐ and solar‐driven CNTs‐based composite PCMs. e) Illustration of the paraffin molecules to CNTs in contact. Reproduced with permission.^[^ ^77^ ^]^ Copyright 2012, American Chemical Society. f) Compressible aligned CNTs arrays and composite PCMs. Reproduced with permission.^[^ ^78^ ^]^ Copyright 2013, American Chemical Society.

Zhang et al.^[^ ^75^ ^]^ also utilized CNTs to prepare SA/CNTs composite PCMs for solar‐to‐thermal conversion. The temperature of SA/CNTs composite PCMs rapidly increased once the solar irradiation is turned on (Figure 9e), which was ascribed to the CNTs, which function as an effective photon captor and molecular heater. To further improve the solar‐to‐thermal conversion efficiency, Qian et al.^[^ ^32^ ^]^ used graphene nanoplatelets (GNP) and CNT hybrids to encapsulate PEG, as shown in **Figure** **10**a. The resulting composite PCMs achieved more than 12‐fold the thermal conductivity of pure PEG, and the corresponding solar‐to‐thermal conversion efficiency reached 86%. This superior comprehensive performance of composite PCMs could be attributed to the difference in filler dimensions.

In addition to functionalized composite PCMs for solar‐to‐thermal conversion and storage, Zheng et al.^[^ ^76^ ^]^ further used CNTs/graphene oxide (GO) hybrid shells to encapsulate long‐chain alkanes (cores) to obtain highly stable and conductive microencapsulated PCMs (MEPCMs) for electric‐to‐thermal conversion (Figure 10b,c). In these microcapsules, multiform CNTs stabilized the capsule shell, thereby resisting the volumetric change‐induced rupture during the heating–cooling cycle process. Moreover, the enhanced thermal conductance helps accelerate expeditious heat exchange. As a result, the working temperature of MEPCMs with a dopant of 5% could be increased by 30% at moderate temperature and low voltage. This design strategy can effectively alleviate the substantial convective heat dissipation from electrothermal system to the surrounding environment.

Chen et al.^[^ ^77^ ^]^ designed a flexible and deformable CNT network for the encapsulation of paraffin, thereby constructing functional composite PCMs for electric‐to‐thermal conversion (Figure 10d). Compared to pure paraffin, these composite PCMs had a higher phase change enthalpy and thermal conductivity, which was attributed to the intermolecular C—H…Π interactions among C—H bonds in the paraffin and delocalized *π* electrons on the surface of the CNTs (Figure 10e). The electric‐to‐thermal conversion efficiency of composite PCMs was ≈40.6% at 1.5 V. However, this electric‐to‐thermal conversion efficiency is relatively lower. To improve the conversion efficiency, Liu et al.^[^ ^78^ ^]^ prepared a compressible and elastic CNT array (CNTA) composed of vertically aligned nanotubes. The CNT density of CNTA could be tailored by direct lateral compression. In their study, *n*‐eicosane (C20) was infiltrated into CNTA to obtain CNTA‐C20 composite PCMs (Figure 10f). The electric‐to‐thermal conversion efficiency of the CNTA‐C20 composite PCMs was 74.7% at 1.3 V. These findings indicated that compressed CNTA could effectively reduce the triggering voltage of the phase change, which accounted for the enhanced heat transfer and reduced bulk resistance in the densified CNTA.

**Figure 11:**
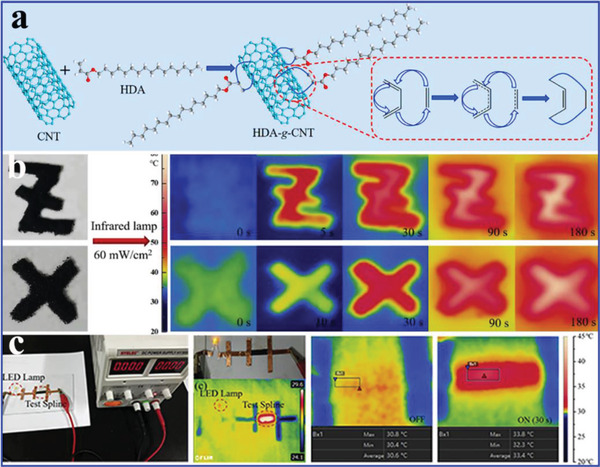
a) Preparation scheme of HDA‐g‐SWCNTs and HDA‐g‐MWCNTs. b) FLIR camera images of HDA‐g‐MWCNTs, and HDA‐g‐SWCNTs. c) Digital photos of the electrical conductivity experimental design and FLIR camera images of HDA‐g‐SWCNT film under 30 V. Reproduced with permission.^[^ ^79^ ^]^ Copyright 2019, Elsevier.

More attractively, Cao et al.^[^ ^79^ ^]^ fabricated hexadecyl acrylate‐functionalized SWCNT and MWCNT (HDA‐g‐SWCNTs and HDA‐g‐MWCNTs) based composite PCMs, which could effectively convert solar and electric energy into thermal energy (**Figure** **11**a). In these composite PCMs, SWCNTs and MWCNTs were the thermally and electrically conductive fillers, and HDA was covalently grafted onto the surface of SWCNTs and MWCNTs via a solvent‐free Diels–Alder (DA) reaction. The obtained HDA‐g‐SWCNTs and HDA‐g‐MWCNTs both exhibited excellent thermal conductivity values of 0.47 and 0.88 W mK^−1^, which are enhanced by 134% and 339% than HAD without CNT‐functionalized, respectively. The corresponding electrical conductivity values were 718 and 389 S m^−1^. Based on the excellent thermal conductivity and electrical conductivity of the HDA‐g‐SWCNTs and HDA‐g‐MWCNTs, the corresponding HDA‐g‐SWCNT and HDA‐g‐MWCNT‐based composite PCMs exhibited efficient solar‐to‐thermal and electric‐to‐thermal conversion efficiency (Figure 11b,c). Moreover, the composite PCMs exhibited good thermal stability, thermal reliability, and shape stability. More importantly, the composite PCMs could absorb the generated thermal energy during the operation of electronic devices, thereby achieving the effective thermal management of electronic devices.

**Figure 12:**
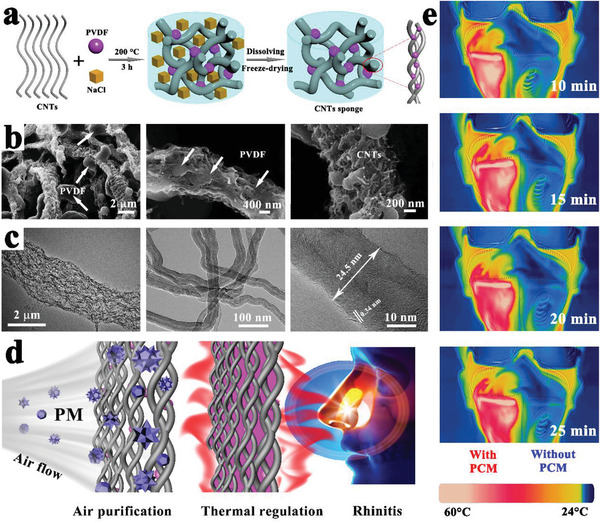
a) Preparation scheme of CNTs sponge. b,c) SEM and TEM images of CNTs sponge. d) Schematic illustration of the thermotherapy. e) Actual thermotherapy effect evaluation (left is a thermotherapy mask, right is a traditional mask). Reproduced with permission.^[^ ^80^ ^]^ Copyright 2020, Elsevier.

Recently, with the rapid development of advanced multifunctional composite PCMs such as the aforementioned solar‐to‐thermal and electric‐to‐thermal conversion PCMs, more innovative features of composite PCMs have been exploited. For example, our group designed advanced flexible hierarchical CNT framework‐based composite PCMs for the high‐performance thermotherapy of allergic rhinitis.^[^ ^80^ ^]^ A 3D freestanding flexible CNT framework was prepared using a sacrificial template (**Figure** **12**a), which was interconnected by PVDF binders and CNT bundles. SEM and transmission electron microscopy (TEM) images (Figure 12b,c) revealed that the PVDF binder played an essential role in the self‐assembly of CNTs into a 3D network through an end‐to‐end joining mechanism.^[^ ^81^, ^82^, ^83^ ^]^ The resulting hierarchical CNT network framework composed of micropores, mesopores, and macropores was considered a compatible supporting host for the encapsulation of PEG. Finally, we designed a functional thermotherapy mask for the thermotherapy of allergic rhinitis, which was composed of an inner CNT framework based composite PCM layer (thermal regulation layer) and an outer pristine CNT sponge layer (air purification layer), as shown in Figure 12d. It is worth noting that the inner CNT framework based composite PCM layer played a dominant role in the thermotherapy process by releasing sustained heat, whereas the outer pristine CNT sponge layer played only a supporting role in the thermotherapy process by purifying inhaled air. The corresponding thermotherapy tests indicated that the thermotherapy mask could continuously provide sufficient hot air flow into the nasal cavity for as long as ≈33 min at ≈43.5 °C, in contrast with an ordinary mask (Figure 12e). To further verify the thermotherapy efficacy, the relevant medical indicators were provided. The experimental evidences further indicated that the thermotherapy mask could sufficiently weaken the inflammatory damage of nasal mucosa.

### 5.2 Carbon Fibers‐Based Composite PCMs

**Figure 13:**
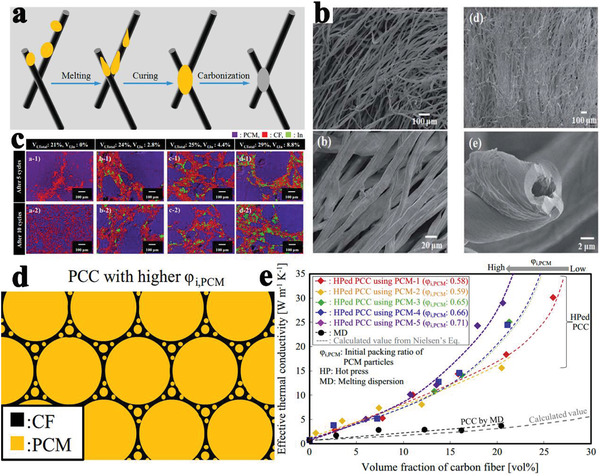
a) Preparation scheme of carbon bonded CFs monolith. Reproduced with permission.^[^ ^84^ ^]^ Copyright 2018, Elsevier. b) SEM images of the original cotton fiber and carbon.^[^ ^15^ ^]^ Copyright 2019, Royal Society of Chemistry. c) EDS of composite PCMs. Reproduced with permission.^[^ ^85^ ^]^ Copyright 2016, Elsevier. d) Simplified relationship model between PCMs and CFs. e) Effective thermal conductivities of composite PCMs prepared by hot‐press method and melt‐mixing method. Reproduced with permission.^[^ ^86^ ^]^ Copyright 2015, Elsevier.

Carbon fibers (CFs) have shown great application potential in the enhancement of the mechanical and thermal properties of composite PCMs, due to their advantageous features, including light weight, high tensile modulus, high thermal conductivity, and dimensional stability. Based on these advantages, Jiang et al.^[^ ^84^ ^]^ prepared carbon‐bonded CFs monoliths from graphite fibers and phenolic resins after carbonization (**Figure** **13**a). The powdered phenolic resins were melted and glided along the fiber surface under gravity, thereby forming junctions to bind the fibers together. After the CFs were infiltrated with paraffin, the resulting composite PCMs exhibited different thermal conductivity values with respect to their directions due to the anisotropy of the CFs. The in‐plane thermal conductivity of composite PCMs was 57 times greater than that of pure paraffin, whereas the out‐of‐plane thermal conductivity of the composite PCMs was 5.5 times greater. It is worth noting that the enhanced thermal conductivity of the composite PCMs exhibited an approximately linear relationship with the volume fraction of CFs. Similarly, Sheng et al.^[^ ^15^ ^]^ fabricated vertically aligned hollow CFs with different densities via the direct carbonization of rolled cotton sheets at 2400 °C (Figure 13b). After paraffin was infiltrated into CFs using a vacuum impregnation method, the anisotropic composite PCMs were successfully prepared. The resulting composite PCMs exhibited good shape stability and anisotropic thermal conductivity due to the interconnected vertically aligned CF framework. The thermal conductivity (0.77 W mK^−1^) of composite PCMs along the axial direction of the aligned CFs was higher than that (0.58 W mK^−1^) along the lateral direction of the aligned CFs.

Deviating from the conventional melt‐dispersion (MD) method, Nomura et al.^[^ ^85^ ^]^ prepared erythritol based composite PCMs using a novel hot‐pressing (HP) method, in which CFs (900 W mK^−1^ in the fiber direction) and indium particles (82.80 W mK^−1^) served as fillers with high thermal conductivity. The melted indium particles welded the CFs to construct a stable percolating network, which improved the thermal conductivity and cyclic endurance of composite PCMs. As shown in Figure 13c, the composite PCMs without indium particles partially collapse after 5 cycles and were destroyed completely after 10 cycles. In contrast, the network structure of composite PCMs with indium particles was maintained even after 10 cycles. The proposed relationship models (Figure 13d) between the PCMs and CFs revealed that the composite PCMs with a higher packing ratio had a special filler configuration, indicating that a high packing ratio could accelerate the formation of the percolating filler network.^[^ ^86^ ^]^ Compared to the conventional MD method, the HP method made it easier to form the percolating filler network in composite PCMs. As a result, when the volume fraction of CFs increased, the thermal conductivity of composite PCMs prepared by HP exponentially increased, while those of composite PCMs prepared by MD gradually increased (Figure 13e).

**Figure 14:**
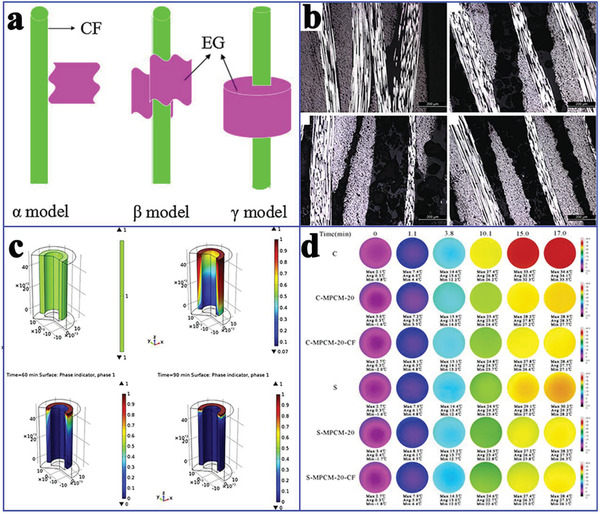
a) The contact types between EG and CFs. Reproduced with permission.^[^ ^87^ ^]^ Copyright 2016, Elsevier. b) Optical images of the laminates. Reproduced with permission.^[^ ^88^ ^]^ Copyright 2018, Elsevier. c) Phase transition process observation at different time. Reproduced with permission.^[^ ^89^ ^]^ Copyright 2016, Elsevier. d) Infrared images of composite PCMs with time. Reproduced with permission.^[^ ^90^ ^]^ Copyright 2018, Elsevier.

Yang et al.^[^ ^87^ ^]^ further employed CFs and EG as thermally conductive fillers to achieve the synergistic enhancement of the thermal conductivity of paraffin‐based composite PCMs. It is worth noting that there will be a risk of CFs agglomeration as the number of CFs increases. The authors proposed three contact types between EG and CFs: point contact, line contact, and face contact (**Figure** **14**a). Different contact types between EG and CFs would construct different thermally conductive surfaces with different thermal resistances. Fredi et al.^[^ ^88^ ^]^ proposed that the thermal conductivity of laminates (Figure 14b) across the thickness is proportional to the content of CF‐reinforced and CNT‐stabilized composite PCMs. Their results showed that the CF‐reinforced flexural modulus was only slightly affected by PCMs, whereas the flexural strength, strain‐at‐break, and interlaminar shear strength showed a decrease, due to the preferential location of PCMs in the interlaminar regions. Karimi et al.^[^ ^89^ ^]^ studied the effect of CFs on the thermal performances of composite PCMs. Their results showed that CF‐enhanced thermal conductivity (155%) made the temperature distribution more uniform within lithium ion battery cells. The interface shapes for different CF loadings were basically the same (Figure 14c). However, the extent of melting region was reduced with increasing the CF mass fraction. In addition, CFs were used as a reinforced alternative to alkali‐activated slag MEPCMs to enhance the mechanical properties. As a result, the compressive strength of CFs‐reinforced composite PCMs was ≈30% higher than that of PCMs without CFs. Furthermore, the thermal transfer capacity was substantially improved through the synergistic enhancement of CFs and graphite (Figure 14d).

### 5.3 Graphene‐Based Composite PCMs

2D graphene, a monolayer crystal of carbon atoms in the form of a hexagonal lattice, exhibits extremely attractive properties, including high‐temperature resistance, strong solar absorption capacity, and high electrical and thermal conductivity.^[^ ^91^, ^92^, ^93^, ^94^, ^95^, ^96^ ^]^ Therefore, 2D graphene is a promising candidate for enhancing the thermal conductivity and photothermal and electrothermal conversion efficiency of composite PCMs. In addition, 2D graphene and its derivatives can be assembled into 3D macroscopic and lightweight structural materials, including graphene foams and graphene aerogels, to further improve the thermal conductivity, photothermal, and electrothermal conversion efficiency of composite PCMs.^[^ ^97^, ^98^, ^99^, ^100^, ^101^, ^102^, ^103^, ^104^, ^105^, ^106^ ^]^

**Figure 15:**
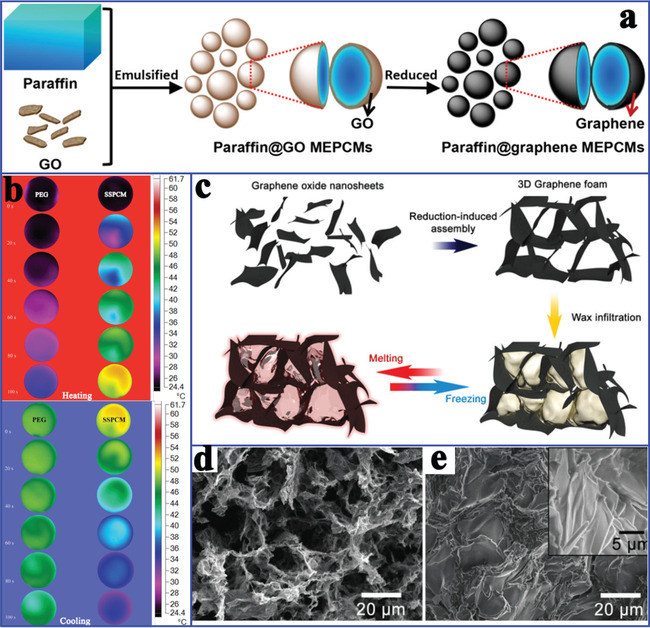
a) Preparation scheme of microencapsulated PCMs. Reproduced with permission.^[^ ^107^ ^]^ Copyright 2018, Springer Nature. b) Thermal infrared images of pure PEG and composite PCMs during the heating and cooling processes. Reproduced with permission.^[^ ^99^ ^]^ Copyright 2029, Elsevier. c) Preparation scheme of the graphene foam and graphene/paraffin composite PCMs. d) SEM image of graphene foam. e) SEM image of graphene/paraffin composite PCMs. Reproduced with permission.^[^ ^109^ ^]^ Copyright 2016, Royal Society of Chemistry.

Considering the inherent ultrahigh thermal conductivity of graphene, Yang et al.^[^ ^107^ ^]^ prepared paraffin/graphene MEPCMs through in situ chemical reduction by adding hydrazine hydrate (**Figure** **15**a). Hot‐pressing molding effectively constructed a segregated structure with a high number of thermal transfer paths in the graphene shell. Therefore, the segregated‐structure composite PCMs integrated high latent heat of 232.4 J g^−1^ and thermal conductivity of 0.42 W mK^−1^, which was 2.34 times that of pure paraffin. Tang et al.^[^ ^108^ ^]^ prepared composite PCMs by in situ filling PEG in a 3D GO network side‐to‐side cross‐linked by Ca^2+^. The obtained composite PCMs exhibited a high latent heat of 218.9 J g^−1^ and the thermal conductivity was enhanced by 87.7% over that of pure PEG. Xia et al.^[^ ^99^ ^]^ used GO‐induced lamellar structures to fabricate composite PCMs (9.0 wt% GO) through self‐assembly and grafted polymerization, which exhibited a latent heat of 158.2 J g^−1^ and thermal conductivity of 0.48 W mK^−1^ with a 41.4% enhancement over that of pure PEG. The improved thermal conductivity obtained by adding GO (Figure 15b) ensured fast thermal response rates and excellent TES characteristics of composite PCMs. Zhang et al.^[^ ^109^ ^]^ constructed 3D graphene foam through the in situ chemical reduction of GO. The obtained paraffin/graphene foam composite PCMs effectively integrated high latent heat of 200 J g^−1^ and thermal conductivity of 0.62 W mK^−1^ due to the 3D interconnected foam structure of graphene (Figure 15c–e).

Yang et al.^[^ ^110^ ^]^ prepared MEPCMs with paraffin core and GO‐modified calcium carbonate (CaCO_3_) shell. The addition of GO could improve the heat storage capacity and thermal stability of MEPCMs. When the content of GO was 1.0 wt %, the encapsulation ratio of MEPCMs reached 73.19%. The thermal conductivity of GO‐modified MEPCMs was 0.88 W mK^−1^ while the thermal conductivity of MEPCMs without GO was 0.72 W mK^−1^. Yang et al.^[^ ^111^ ^]^ also prepared MEPCMs with paraffin core and melamine‐formaldehyde resin shell. GO nanosheets were situated at the interface between the core and the shell. MEPCMs with 0.5 mg mL^−1^ GO had a high encapsulation ratio of 93.9 wt% paraffin, and their leakage rate was reduced by 93.1% compared with that of MEPCMs without GO. In addition to organic composite PCMs, Tao et al.^[^ ^70^ ^]^ prepared four types of carbon‐based inorganic composite PCMs with Li_2_CO_3_‐K_2_CO_3_ binary carbonate eutectic salts as PCMs. Compared with C60, SWCNTs, and MWCNTs, graphene was the best additive to improve the specific heat (18.57%). The greater the specific surface area is, the greater the surface energy is. A larger surface energy can promote the formation of nanolayers that have a positive effect on improving the specific heat, and accelerate the aggregation of nanomaterials that have a negative effect on improving specific heat.

**Figure 16:**
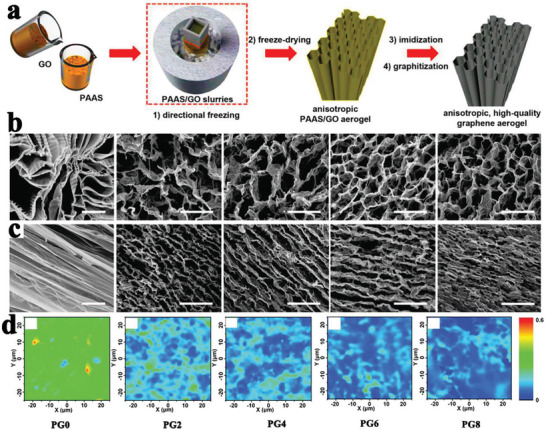
a) Preparation scheme of AGAs. b) Longitudinal view SEM images. c) Transversal view SEM images. d) Raman mappings of AGAs. Reproduced with permission.^[^ ^112^ ^]^ Copyright 2018, Wiley‐VCH.

Similar to the CNT distribution mentioned earlier, an arrayed graphene distribution usually has more advantages than a random graphene distribution in terms of the thermal conductivity enhancement of PCMs. According to this arrayed design guide, Min et al.^[^ ^112^ ^]^ adopted anisotropic graphene aerogels (AGAs) by directionally freezing aqueous suspensions of polyamic acid salt and GO, followed by imidization at 300 °C and graphitization at 2800 °C (**Figure** **16**a). After adding GO, a distinct anisotropic structure and lamellar‐to‐tubular transition were clearly observed in both the longitudinal and the transverse directions (Figure 16b,c). Several lamellae were arranged in parallel for constructing short‐range lamellar domains, which were mutually connected at various crosslinking points. Moreover, AGAs exhibited a higher specific surface area and graphitization degree (Figure 16d) as the GO concentration increases. Therefore, GO played an important role in designing high‐quality AGAs. After infiltrating paraffin, the thermal conductivity values of the resultant paraffin/AGAs composite PCMs were 2.68 W mK^−1^ along the transverse direction and 8.87 W mK^−1^ along the longitudinal direction, which was ≈24 times higher than that of pure paraffin. This outstanding thermal conductivity was originated from the arrayed structure and high specific surface area of AGAs.

**Figure 17:**
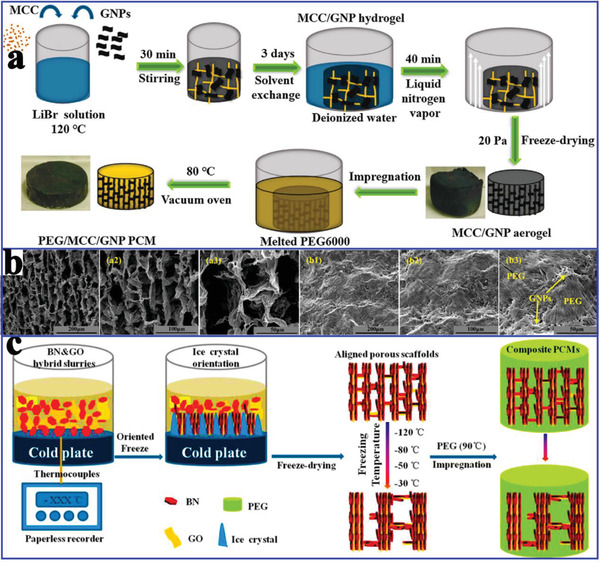
a) Preparation scheme of MCC/PEG/GNP composite PCMs. b) SEM images of MCC/GNP‐1.51 aerogel and MCC/PEG/GNP‐1.51 composite PCMs. Reproduced with permission.^[^ ^100^ ^]^ Copyright 2019, Elsevier. c) Preparation scheme of the hybrid porous scaffolds and composite PCMs. Reproduced with permission.^[^ ^113^ ^]^ Copyright 2018, American Chemical Society.

In addition to the individual use of graphene for the thermal conductivity enhancement of PCMs, Wei et al.^[^ ^100^ ^]^ prepared arrayed hybrid cellulose/GNP aerogel through solution compounding, gelling, and solvent exchange (**Figure** **17**a). The obtained 3D arrayed framework could be clearly observed in Figure 17b. It is worth noting that increasing GNP could facilitate the anisotropic structure of cellulose/GNP. After PEG was infiltrated into the arrayed cellulose/GNP, the obtained cellulose/GNP/PEG composite PCMs exhibited a high latent heat (182.6 J g^−1^) and high thermal conductivity (1.03 W mK^−1^) at a very low GNP content (1.51 wt%). The thermal conductivity was 232% higher than that of the PEG/cellulose composite PCMs without GNP. This significant thermal conductivity enhancement originated from the arrayed and 3D segregated structure of cellulose/GNP, which could guarantee the homogeneous distribution of PEG molecules in thermally conductive paths.

Additionally, Yang et al.^[^ ^114^ ^]^ further used hybrid graphene aerogel and graphene foam to encapsulate paraffin for preparing composite PCMs with a high thermal conductivity of 1.82 W mK^−1^, which was 574% higher than that of pure paraffin. This excellent thermal conductivity stems from the additional thermally conductive pathways of the graphene aerogel/foam, which contributes to the obvious reduction of the interfacial thermal resistance and acceleration of the thermal transport rate. Yang et al.^[^ ^113^ ^]^ also prepared arrayed GO/boron nitride (BN) hybrid porous scaffold using a unidirectional ice‐templated strategy (Figure 17c). Interestingly, adjusting the freezing temperature could construct various thermally conductive pathways. The optimal thermal conductivity of the PEG/GO/BN composite PCMs was 3.18 W mK^−1^ at 28.7 wt% BN loading, which was 864% higher than that of pure PEG.

**Figure 18:**
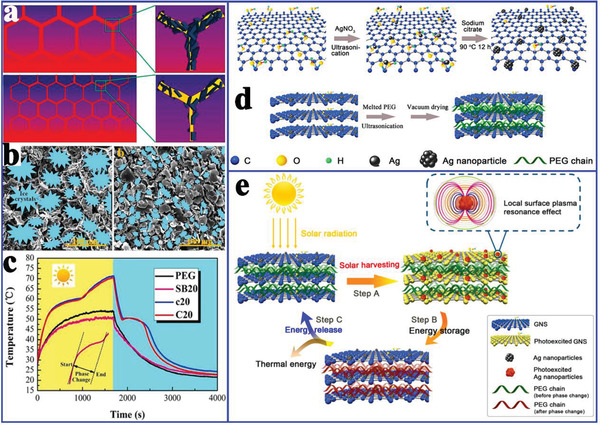
a) Schematic diagram of the structure and mechanism of different thermal conductivity effects. b) The formation process of 3D porous scaffolds. c) Solar‐to‐thermal energy conversion curves of PEG and composite PCMs. Reproduced with permission.^[^ ^115^ ^]^ Copyright 2016, Royal Society of Chemistry. d) Preparation scheme of Ag‐GNS and Ag‐GNS/PEG composite PCMs. e) Schematic illustration of the solar‐to‐thermal energy conversion. Reproduced with permission.^[^ ^106^ ^]^ Copyright 2019, Elsevier.

In addition to exhibiting excellent thermal storage and thermal conduction properties, other advanced functions of composite PCMs are also necessary. Therefore, Yang et al.^[^ ^115^ ^]^ prepared GO/BN hybrid porous scaffold based composite PCMs using an ice‐templated assembly strategy for solar‐to‐thermal conversion and storage. The resultant composite PCMs showed a high thermal storage density (145.9 J g^−1^) and thermal conductivity of 1.84 W mK^−1^ at 19.2 wt% BN loading, which was much higher than that of composite PCMs obtained by the solution blending method. This higher thermal conductivity was attributed to the self‐assembly of the thermally conductive fillers during ice growth. It is worth mentioning that a lower freezing temperature could generate more ice crystal nuclei and thinner ice fingers, thereby forming more effective heat transfer paths (**Figure** **18**a,b). Furthermore, the composite PCMs could contribute to efficient solar‐to‐thermal energy conversion and storage (Figure 18c) due to the effective photon capturing ability of GO/BN. To further enhance the visible‐light absorption and photothermal conversion of composite PCMs, Zhang et al.^[^ ^106^ ^]^ reported Ag nanoparticle‐functionalized graphene nanosheets (Ag‐GNS) based composite PCMs with a high thermal storage density (>166.1 J g^−1^), enhanced thermal conductivity (95.3%), and a high solar‐to‐thermal conversion efficiency of 92.0% (Figure 18d,e). This excellent comprehensive thermal performance was attributed to the synergistic effect of Ag and GNS.

**Figure 19:**
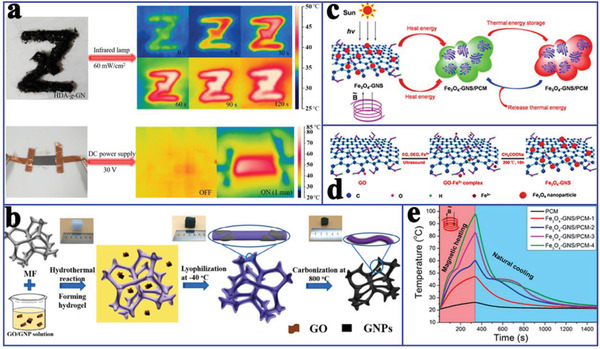
a) Thermal infrared images of HDA‐g‐GN under an infrared lamp and DC power supply. Reproduced with permission.^[^ ^116^ ^]^ Copyright 2019, American Chemical Society. b) Preparation scheme of the MF‐templated hybrid aerogels. Reproduced with permission.^[^ ^98^ ^]^ c) Scheme of the magnetic‐ and solar‐to‐thermal energy conversion and storage. d) Formation mechanism of Fe_3_O_4_‐GNS. e) Magnetic‐to‐thermal energy conversion curves (1.36 MHz and 550 A m^−1^). Reproduced with permission.^[^ ^34^ ^]^ Copyright 2017, Royal Society of Chemistry.

In addition to functionalized composite PCMs for only solar‐to‐thermal energy conversion, Cao et al.^[^ ^116^ ^]^ prepared hexadecyl acrylate‐grafted graphene (HDA‐g‐GN) via a solvent free Diels‐Alder reaction for both solar‐to‐thermal and electric‐to‐thermal energy conversion. The resultant composite PCMs exhibited a high thermal conductivity (3.96 W mK^−1^) and electrical conductivity (219 S m^−1^). More importantly, the composite PCMs also demonstrated solar‐to‐thermal and electric‐to‐thermal energy conversion capacity (**Figure** **19**a). As shown in Figure 19a, the patterned letter “z” with HDA‐g‐GN became increasingly brighter with increasing irradiation time. Similarly, the temperature of HDA‐g‐GN increased rapidly to 71.1 °C for 1 min at 30 V. Li et al.^[^ ^9^ ^]^ prepared AGA‐based composite PCMs for both solar‐to‐thermal and electric‐to‐thermal energy conversion. The resulting composite PCMs were triggered not only by the weak solar irradiation (0.8–1.0 sun) with a solar‐to‐thermal conversion efficiency of up to 77.0%, but also by a small voltage (1–3 V) with an electric‐to‐thermal conversion efficiency of up to 85.4%. The excellent solar‐to‐thermal conversion capacity of graphene aerogel‐based composite PCMs originated from the generated microcavities among graphene‐paraffin‐graphene sandwich junctions. More importantly, the critical voltage for driving and completing phase change could be as low as 1.0 V.

To further improve the energy conversion efficiency of composite PCMs, Xue et al.^[^ ^98^ ^]^ utilized the commercial melamine foam (MF) incorporating GO and GNP (Figure 19b) to fabricate paraffin/rGO/GNP/MF composite PCMs. When the filler content was 4.89 wt%, the composite PCMs exhibited a high thermal conductivity (1.46 W mK^−1^), electrical conductivity (2.79 S cm^−1^), and phase change enthalpy retention rate (nearly 100% that of paraffin). This excellent performance originated from the coverage of the conductive rGO/GNP filler on the carbonized MF framework and the shrinkage of the aerogel during the carbonization process. As a result, the surface temperature of composite PCMs was higher than that of pure paraffin at the same heating time, indicating that the composite PCMs had a faster heat diffusion rate. In addition, the paraffin/rGO/GNP/MF composite PCMs also exhibited excellent solar‐to‐thermal energy conversion efficiency (88%) and electric‐to‐thermal energy conversion efficiency (62.5%). These indicated that the composite PCMs may be utilized for heat preservation in buildings or thermal protection in microelectronic devices.

More attractively, Wang et al.^[^ ^34^ ^]^ introduced Fe_3_O_4_‐functionalized GNS (Fe_3_O_4_‐GNS) to fabricate magnetic‐ and solar‐ driven energy conversion and storage PCMs (Figure 19c). Fe^3+^ was captured by hydroxyl, carboxyl, or epoxy groups on the GO by coordination and was partially reduced to Fe^2+^ during the solvothermal treatment, thereby forming Fe_3_O_4_ nanoparticles on the reduced GNS (Figure 19d). Importantly, the Fe_3_O_4_‐GNS‐based composite PCMs exhibited a high solar‐to‐thermal energy conversion efficiency of 92.3% due to the strong solar capture capability of GNS. This superior solar‐to‐thermal conversion efficiency of Fe_3_O_4_‐GNS/PEG composite PCMs was comparable to that of SWCNT‐based composite PCMs (91.3%)^[^ ^117^ ^]^ and dye‐grafted composite PCMs (93.7%).^[^ ^118^ ^]^ In addition, an alternating magnetic field directly triggered magnetic‐to‐thermal conversion of Fe_3_O_4_‐GNS/PEG composite PCMs (Figure 19e) due to the magnetothermal effect of Fe_3_O_4_ nanoparticles.^[^ ^105^, ^119^ ^]^ The corresponding magnetic‐to‐thermal energy conversion efficiency was 41.7%. It is worth noting that the magnetic‐to‐thermal conversion efficiency could be further improved by increasing Fe_3_O_4_‐GNS content in the composites and improving the utilization rate of the alternating magnetic field.

**Figure 20:**
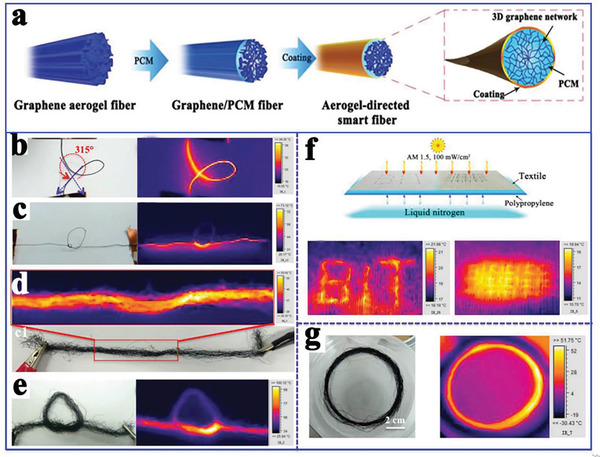
a) Preparation scheme of the graphene/PCMs smart fibers. b–e) Photograph and IR images of the aerogel‐directed smart fibers under an input voltage of 30 V. f,g) Photograph and IR images of the aerogel‐directed smart fibers under solar irradiation of 1.0 sun. Reproduced with permission.^[^ ^120^ ^]^ Copyright 2018, Wiley‐VCH.

To take advantage of the intelligent and integrative functions of composite PCMs in response to multiple external stimuli, Li et al.^[^ ^120^ ^]^ prepared advanced multiresponsive PEG@graphene aerogel phase change smart fibers coated with a hydrophobic fluorocarbon resin (**Figure** **20**a), which exhibited a wide range of phase change enthalpies (0–186 J g^−1^) and temperatures. These strong and compliant phase change smart fibers could be twisted into yarn and woven into fabrics, which smartly responded to multiple external stimulius signals (thermal, electrical, and photonic) and exhibited reversible thermal energy conversion and storage. In the IR images of the photonic response, the higher temperature distribution mostly emerged along a straight line through the knots (Figure 20b), which might be due to the improved thermal and electrical conductivity caused by the compressed and twisted graphene network (Figure 20c). This observation indicated that the self‐twined knots had a lower interfacial contact resistance. It is worth noting that the superhydrophobic coating could accelerate electron transfer between fibers through electron tunneling. The temperature of the normal fiber bundles reached 56 °C (Figure 20d) at 30 V, whereas the knotted fiber bundles reached a higher temperature of 100 °C (Figure 20e). In addition, the fibers could also respond to the solar‐to‐thermal conversion in a low temperature environment (0 °C), as shown in Figure 20f,g. Furthermore, the superhydrophobic fluorocarbon coating rendered the fibers waterproof, and their self‐cleaning features further promote their mechanical properties. Therefore, the PEG/graphene aerogel smart fibers showed great application potential for future flexible and wearable devices. In addition, Kim et al.^[^ ^121^ ^]^ developed a smart release system responsive to near‐infrared (NIR) light by coencapsulating rGO, branched polyethylenimine, PEG, doxorubicin (DOX) (an anticancer drug), and glutathione for intracellular drug delivery. The effective release of the loaded DOX could be realized by regulating the NIR photothermal effect and glutathione.

### 5.4 MOFs‐Derived Carbon‐Based Composite PCMs

Metal organic frameworks (MOFs) are emerging 3D porous organic–inorganic hybrid materials that are synthesized by the self‐assembly of organic ligands and metal ions/metal clusters.^[^ ^122^, ^123^, ^124^, ^125^ ^]^ They are promising candidates for preparing shape‐stabilized composite PCMs because of their superior advantages, including diverse structural topologies, adjustable pore sizes, controllable surface properties, ultrahigh surface area, high porosity, and stable thermochemical properties.^[^ ^52^, ^126^, ^127^, ^128^ ^]^ Our group first introduced MOFs as the supporting materials to obtain shape‐stabilized composite PCMs.^[^ ^47^ ^]^ The influences of different pore sizes and functional groups of MOFs (Zn: MOF‐5, IRMOF‐3; Zr: UiO‐66, UiO‐66‐NH_2_; Al: MIL‐53, MIL‐53‐NH_2_; Cr: MIL‐101, MIL‐101‐NH_2_) on the thermal storage performances of organic PCMs had been systematically studied. Interestingly, amino‐modified MOFs exhibited a higher loading content of PCMs and phase change enthalpy than the corresponding pristine MOFs. The enhancement mechanism is that amino functional groups provided more chemical adsorption sites of PCMs and secondary interactions by the hydrogen bonding between PCMs and amino functional groups.^[^ ^129^ ^]^ However, their thermal enthalpies were significantly lower than the theoretical values. Surprisingly, SA/MOF‐5 composite PCMs exhibited nearly no latent heat (3.8 J g^−1^) due to the strong confinement effect induced by the small pore size of MOF‐5.

**Figure 21:**
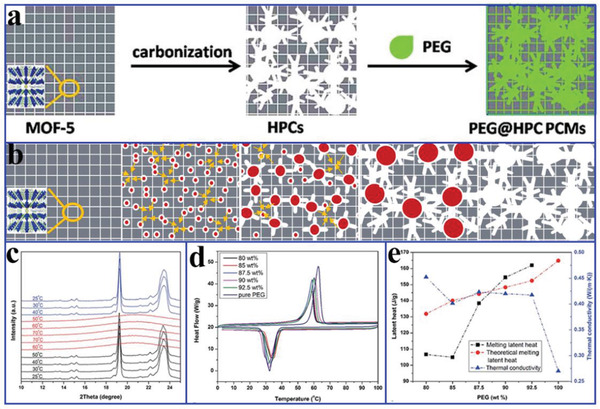
a) Preparation diagram of PEG/HPC composite PCMs. b) Formation mechanism of highly hierarchical porous carbon. c) XRD patterns of PEG/HPC‐1000 composite PCMs at various temperatures. d) DSC curves. e) Thermal conductivities and latent heats of pure PEG and PEG/HPC‐1000 composite PCMs. Reproduced with permission.^[^ ^130^ ^]^ Copyright 2016, Royal Society of Chemistry.

To solve the strong confinement effect of MOF‐5 on PCMs, Tang et al.^[^ ^130^ ^]^ carbonized MOF‐5 into nanoporous carbon (**Figure** **21**a). During calcination, tiny nanoparticles first emerged, and then migrated and aggregated into larger particles. Finally, ZnO particles of different sizes were evaporated to construct a hierarchical porous carbon (HPC) structure composed of micropores, mesopores, and macropores (Figure 21b). The pore size of MOF‐5‐derived nanoporous carbon (4.4 nm) was much larger than that of pristine MOF‐5 (1.3 nm). After carbonization, the BET surface area and pore volume increased to 2804 m^2^ g^−1^ and 3.11 cm^3^ g^−1^, respectively (1060 m^2^ g^−1^ and 0.34 cm^3^ g^−1^ for MOF‐5). The pore surface properties also changed. The experimental results indicated that MOF‐5 derived nanoporous carbon with a PEG4000 content of up to 92.5 wt% had a high melting enthalpy of 162 J g^−1^ (164.9 J g^−1^ for pure PEG4000). The crystallization fraction could reach 98.2% due to the free phase change behaviors of PEG4000 in the enlarged hierarchical pores (Figure 21c–e). It can be concluded that MOF‐5 derived HPC provided a good solution to the strong nanoconfinement effect of pristine MOF‐5 on PCMs. However, the thermal conductivity of the corresponding composite PCMs was only 0.42 W mK^−1^ (0.27 W mK^−1^ for pure PEG4000). Although MOF‐5 derived nanoporous carbon could efficiently stabilize PCMs and solve the leakage issue, the low thermal conductivity still limited their practical applications.

**Figure 22:**
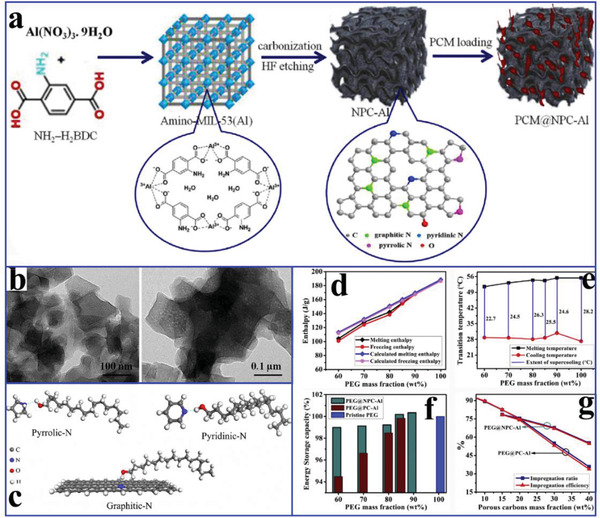
a) Preparation diagram of PEG/NPC‐Al composite PCMs, b) TEM images of NPC‐Al. c) Schematic diagram of hydrogen bond interactions between doped nitrogen and PCMs molecules. d) Actual and calculated latent heat values. e) Supercooling characteristics. f) TES capabilities. g) Impregnation characteristics of composite PCMs. Reproduced with permission.^[^ ^131^ ^]^ Copyright 2019, Elsevier.

To enhance the thermal conductivity of MOFs‐derived nanoporous carbon‐based composite PCMs, Atinafu et al.^[^ ^131^ ^]^ synthesized N‐doped porous carbon (NPC‐Al) to prepare PEG‐based composite PCMs by carbonizing Al‐MIL‐53‐NH_2_ (**Figure** **22**a,b). NPC‐Al exhibited a large BET of 2193.5 m^2^ g^−1^, high mesopore proportion and high nitrogen content, which was difficult to obtain via postsynthesis. The authors systematically studied the effect of doped nitrogen on the loading content, phase change enthalpy, thermal storage efficiency, and thermal conductivity. Interestingly, NPC‐Al exhibited a high loading PEG of 90 wt%, a thermal storage capability of 100.3% and an enhanced thermal conductivity of 52% (Figure 22d–g), which was higher than that of carbon without nitrogen doping obtained from the same calcination process. Different nitrogen atoms (pyrrolic‐N, graphitic‐N, and pyridinic‐N) played important roles in the adsorption, distribution and stabilization of PEG via hydrogen bonding (Figure 22c).^[^ ^132^, ^133^ ^]^ Importantly, a high content of homogeneous graphitic‐N was conducive to accelerating the phonon transmission and improving the thermal conductivity of PCMs.^[^ ^24^, ^131^, ^134^ ^]^

**Figure 23:**
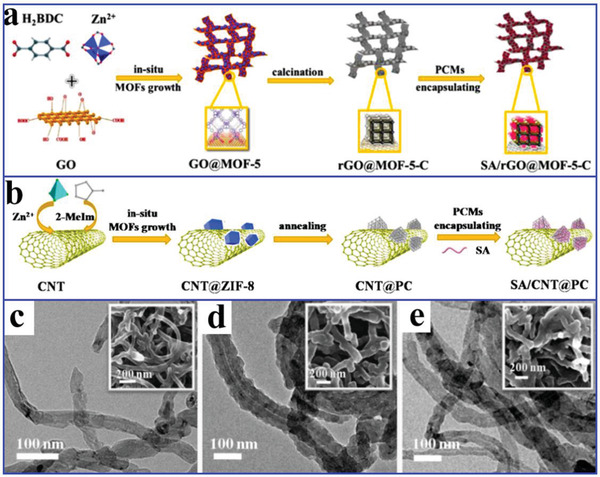
a) Preparation diagram of SA/rGO/MOF‐5‐C composite PCMs. Reproduced with permission.^[^ ^27^ ^]^ Copyright 2018, American Chemical Society. b) Preparation diagram of SA/CNTs/PC composite PCMs. TEM and SEM images: c) CNTs, d) CNTs/ZIF‐8, e) CNTs/PC. Reproduced with permission.^[^ ^135^ ^]^ Copyright 2018, Elsevier.

Although MOFs‐derived nitrogen doped nanoporous carbon can promote the thermal conductivity of composite PCMs, the enhancement effect remains insufficient. To further improve the thermal conductivity of MOFs‐derived nanoporous carbon‐based composite PCMs, Li et al.^[^ ^27^ ^]^ carbonized a GO/MOF‐5 template to synthesize 3D HPC (**Figure** **23**a), thereby simultaneously obtaining excellent thermal storage and thermal conductivity. During the carbonization process, GO was reduced to rGO and MOF‐5 was converted into HPC. SA could be well shape‐stabilized into the obtained 3D HPC through capillary force and surface tension.^[^ ^99^, ^100^, ^101^, ^102^, ^103^, ^104^, ^105^, ^106^, ^107^, ^108^, ^109^, ^110^, ^111^, ^112^, ^113^, ^114^, ^115^, ^116^, ^117^, ^118^, ^119^, ^120^, ^121^, ^122^, ^123^, ^124^, ^125^, ^126^, ^127^, ^128^, ^129^, ^130^, ^131^, ^132^, ^133^, ^134^, ^135^, ^136^, ^137^ ^]^ The interconnected 3D network structure provided sufficient space to freely stretch and crystallize SA molecules and continuous channels for phonon transfer.^[^ ^24^ ^]^ In addition, the interactions between SA and rGO considerably reduced the interfacial thermal resistance, thereby accelerating the transmission of phonons. As a result, the loading content of PCMs reached 90 wt % with a high latent heat of 168.7 J g^−1^ and a crystallization degree of ≈95.6%. Compared with pure SA (0.34 W mK^−1^), the rGO/MOF‐5‐C/SA composite PCMs exhibited a much higher thermal conductivity (0.60 W mK^−1^).

**Figure 24:**
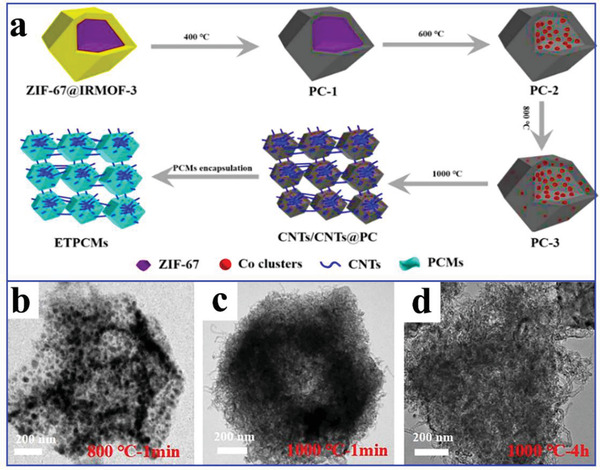
a) Preparation diagram of electric‐to‐thermal composite PCMs. b–d) TEM images of ZIF‐67/IRMOF‐3. Reproduced with permission.^[^ ^138^ ^]^ Copyright 2020, Wiley‐VCH.

Li et al.^[^ ^135^ ^]^ prepared core‐sheath structural CNTs/PC via the in situ carbonation of CNTs/ZIF‐8, in which CNTs served as the core and PC derived from carbonized ZIF‐8 served as the sheath (Figure 23b–e). Interpenetrating CNTs network structure serving as thermal transfer pathways offered continuous channels for phonon transmission.^[^ ^24^ ^]^ The interactions between PCMs and PC/CNTs also reduced the interfacial thermal resistance. The resulting SA/CNTs/PC composite PCMs exhibited a high thermal conductivity of 1.02 W mK^−1^, a high phase change enthalpy of 155.7 J g^−1^ and a high thermal storage capability of 99.9%. Based on this interesting network structure, CNTs‐penetrated porous network carbon was successfully prepared via a gradient carbonization of ZIF/MOFs template (**Figure** **24**a).^[^ ^138^ ^]^ The composite PCMs were triggered by a low voltage of 1.1 V and realized highly efficient electric‐to‐thermal conversion (94.5%). The superior electric‐to‐thermal conversion efficiency was attributable to the following three factors: highly interpenetrating electric/thermal conductive channels facilitating interfacial interactions, the 3D network array structure reducing resistivity, and the low thermal conductive protector decelerating convective heat dissipation (Figure 24b–d).

### 5.5 Biomass‐Derived Carbon‐Based Composite PCMs

To optimize the thermal performance of carbon‐based composite PCMs and achieve the goal of economic and environmental protection,^[^ ^54^, ^78^, ^139^, ^140^ ^]^ simple carbonization strategies with biomass materials have shown promising results, as these materials can be transformed into PC materials.^[^ ^78^, ^141^, ^142^ ^]^ Such biomass‐derived PC materials usually exhibit structural diversity, such as globular,^[^ ^143^, ^144^ ^]^ fibrous,^[^ ^145^, ^146^ ^]^ layered,^[^ ^147^, ^148^ ^]^ and 3D structures.^[^ ^149^, ^150^ ^]^ Large numbers of mesopores and macropores in this carbonized biomass are beneficial for the encapsulation of PCMs.^[^ ^2^, ^24^, ^46^, ^47^, ^55^ ^]^ Moreover, biomass‐derived carbon usually retains an interpenetrating network structure, which is conducive to heat transfer and diffusion, thereby improving the thermal conductivity of composite PCMs.^[^ ^151^ ^]^

**Figure 25:**
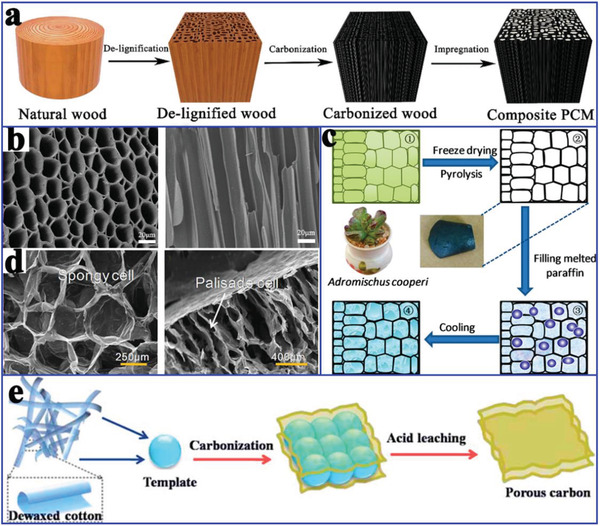
a) Preparation diagram of wood‐derived carbon‐based composite PCMs. b) SEM images. Reproduced with permission.^[^ ^157^ ^]^ Copyright 2018, Royal Society of Chemistry. c) Preparation diagram of adromischus cooperi‐derived carbon‐based composite PCMs. d) SEM images of spongy cell and palisade cell. Reproduced with permission.^[^ ^159^ ^]^ Copyright 2019, Elsevier. e) Preparation diagram of cotton‐derived porous carbon. Reproduced with permission.^[^ ^137^ ^]^ Copyright 2018, Royal Society of Chemistry.

After carbonization, delignified wood (DW) is a promising material for the encapsulation of PCMs owing to the regular pore structure,^[^ ^152^ ^]^ large Brunauer–Emmett–Teller (BET) surface area,^[^ ^9^ ^]^ excellent chemical stability,^[^ ^153^ ^]^ abundant pores,^[^ ^154^ ^]^ nontoxicity, and low‐density.^[^ ^155^ ^]^ Considering these advantages, Montanari et al.^[^ ^156^ ^]^ prepared PEG/DW composite PCMs with a latent heat of 76 J g^−1^. Although an increased PEG content could improve the latent heat of composite PCMs, the mechanical performance was also influenced. To further increase the latent heat, Yang et al.^[^ ^157^ ^]^ prepared the regular porous carbonized woods (PCWs) from sycamore wood through a delignification and carbonization process (**Figure** **25**a,b). The resulting lauric acid (LA) based composite PCMs exhibited a high latent heat of 178.2 J g^−1^.

Succulents that store large amounts of water in their thick flesh have been considered as promising precursors for obtaining 3D PC via dehydration and carbonization.^[^ ^158^, ^159^ ^]^ Wei et al.^[^ ^159^ ^]^ prepared carbon aerogel (CA)‐based composite PCMs from *Adromischus cooperi* via a dehydration and pyrolysis process, followed by the encapsulation of paraffin using a vacuum infusion method (Figure 25c). The encapsulation ratio of paraffin reached 95% in the CA and the resulting composite PCMs exhibited a high melting enthalpy of 133.1 J g^−1^ and a solidifying enthalpy of 147.7 J g^−1^. The closed‐cell structure and two protective layers (Figure 25d) derived from palisade cells and dense epidermal cells guaranteed the excellent leak‐proof performance of composite PCMs.

However, the aforementioned PC materials derived from wood and *A. cooperi* based composite PCMs usually exhibit a lower actual latent heat than expected.^[^ ^160^, ^161^ ^]^ Consequently, Atinafu et al.^[^ ^137^ ^]^ used dewaxed cotton with a cellulose content of 90–95% to fabricate PC with a large specific surface area of 876.6 m^2^ g^−1^ using a Mg(OH)_2_ template (Figure 25e). The resulting composite PCMs exhibited a very high TES density of 219.4 J g^−1^ with an encapsulation ratio of 90 wt%, which approached the theoretical latent heat. Moreover, the composite PCMs demonstrated an enhanced thermal conductivity of 0.40 W mK^−1^ due to a certain degree of graphitization of the cotton‐derived PC.^[^ ^24^, ^131^, ^134^ ^]^

**Figure 26:**
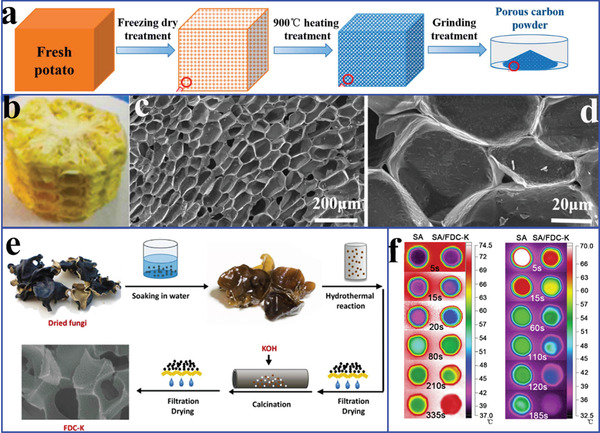
a) Preparation diagram of potato‐derived porous carbon. Reproduced with permission.^[^ ^160^ ^]^ Copyright 2019, Elsevier. b) Photo of corn cob. c,d) SEM images of carbonized corn cob. Reproduced with permission.^[^ ^166^ ^]^ Copyright 2014, Elsevier. e) Preparation diagram of FDC‐K. f) Thermal infrared images of SA and SA/FDC‐K. Reproduced with permission.^[^ ^168^ ^]^ Copyright 2016, Wiley‐VCH.

Agricultural byproducts have also been extensively investigated to prepare PC materials because of their unique cell structures and low cost.^[^ ^162^, ^163^ ^]^ Among them, potato is a promising candidate for the preparation of PC materials due to their porous cellular structure^[^ ^158^, ^164^ ^]^ and high mass percentage of starch.^[^ ^165^ ^]^ Tan et al.^[^ ^160^ ^]^ carbonized fresh potato to prepare hierarchical carbon ranging from several nanometers to several micrometers with a porosity of 73.4% and BET surface area of 42.6 m^2^ g^−1^ (**Figure** **26**a). The corresponding composite PCMs exhibited a latent heat of 91.8 J g^−1^. Zhang et al.^[^ ^166^ ^]^ fabricated a PC derived from corn cobs through freeze drying and carbonization (Figure 26b–d). After the introduction of lauric‐stearic acid (LA‐SA) into the PC, the composite PCMs exhibited excellent melting and freezing latent heats of 148.3 and 144.2 J g^−1^, respectively. The thermal conductivity of composite PCMs was 0.44 W mK^−1^, which was 87.5% higher than that of LA‐SA.

In addition to the popular plant‐derived PC sources, fungi‐derived carbon (FDC) has been reported as a supporting material for TES.^[^ ^167^ ^]^ Li et al.^[^ ^168^ ^]^ fabricated FDC by calcining a soaked fungus with KOH in Ar atmosphere at 800 °C (Figure 26e). For the comparison, dry fungus and hydrothermal charcoal were calcined under the same preparation conditions without KOH. After SA was loaded into the FDC by vacuum‐assisted impregnation, the obtained composite PCMs were denoted as FDC‐K. The melting and freezing enthalpies of FDC‐K were 144.8 and 142.6 J g^−1^, respectively. The thermal conductivity of FDC‐K was 1.15 times higher than that of pure SA. Therefore, FDC‐K had a faster temperature response and more effective heat transfer under the same conditions (Figure 26f).

**Figure 27:**
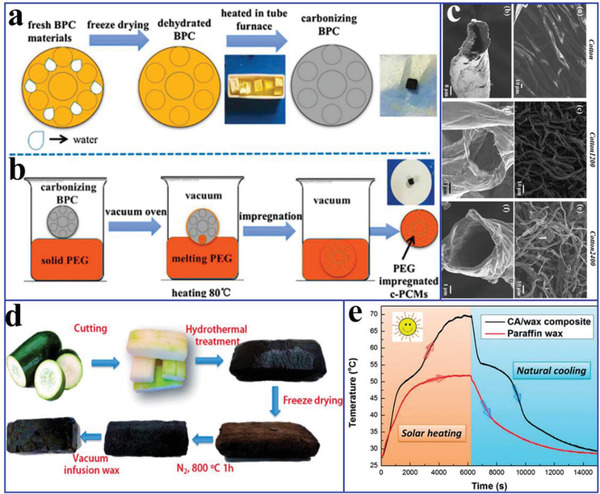
a) Preparation diagram of biological porous carbon. b) Preparation diagram of composite PCMs. Reproduced with permission.^[^ ^161^ ^]^ Copyright 2018, Elsevier. c) SEM images of the original cotton fibers and carbon sponges. Reproduced with permission.^[^ ^171^ ^]^ Copyright 2019, Elsevier. d) Preparation diagram of winter melon derived CAs and CAs/paraffin composite PCMs. e) Solar‐to‐thermal conversion curves of CAs/paraffin composite PCMs. Reproduced with permission.^[^ ^158^ ^]^ Copyright 2014, Royal Society of Chemistry.

In terms of biomass‐derived PC, carbonization temperature plays an important role in the structure and thermal property regulation of composite PCMs. However, the typical carbonization temperature of the biomass‐derived PC, including the aforementioned biomass‐derived PC materials, is usually below 1000 °C. Therefore, higher carbonization temperature studies are necessary because higher temperatures usually stimulate a higher graphitization of carbon. Zhao et al.^[^ ^161^ ^]^ selected potatoes and white radishes as precursors to prepare PC (**Figure** **27**a). It is interesting to note that the sizes of the honeycomb‐like structure were expanded from 50 to 100 µm and that the crystalline structure and graphitization degree were improved as the temperature increases. However, the cellular structure was destroyed when the temperature exceeded 1600 °C. Therefore, the optimal calcination temperature was 1300 °C and the corresponding encapsulation ratio of PEG reached 85.4 wt% (Figure 27b). The melting and freezing enthalpies of composite PCMs were 175.6 and 158.5 J g^−1^, respectively. More importantly, the thermal conductivity of composite PCMs was increased to 4.50 W mK^−1^, which was a tenfold improvement compared to that of pristine PEG. This enhancement was attributed to the interconnected 3D honeycomb‐like carbon heat conduction paths.^[^ ^169^, ^170^ ^]^

To obtain more optimized biomass derived carbon materials at higher temperatures, Sheng et al.^[^ ^171^ ^]^ directly carbonized the sustainable biomass cotton at 1200 and 2400 °C. As a result, the original hollow fibers were retained, and tended to be more curved (Figure 27c). The carbon obtained at 1200 °C showed the typical hard carbon structure with randomly oriented turbostratic nanodomains. The carbon obtained at 2400 °C showed an enhanced graphitization with many fringes corresponding to the graphite (002) plane. Moreover, the hollow CFs were flexible and pressable, indicating that they could be easily used to prepare composite PCMs with desired structures. The encapsulation ratio of paraffin reached 95.5 wt% and the phase change enthalpy of composite PCMs reached 209.3 J g^−1^. Comparatively, the carbon obtained at 2400 °C (0.43 W mK^−1^) provided a greater thermal conductivity than the carbon obtained at 1200 °C (0.31 W mK^−1^), which was attributed to better graphitization at a higher temperature. The authors further investigated the effect of vertical alignment on the thermal conductivity. The thermal conductivity in the axial direction along the fibers was 0.77 W mK^−1^ (>3 times than that of pure paraffin), which was ≈1.33 times that in the lateral direction. This enhanced thermal conduction was attributed to the vertically aligned CFs.^[^ ^15^ ^]^

To satisfy the multifunctional needs of composite PCMs beyond TES and transfer, Yang et al.^[^ ^172^ ^]^ reported multifunctional DW based composite PCMs with a latent heat of 119.2 J g^−1^ by mixing thermochromic (TC) compounds, bisphenol A(BPA), and 1‐tetradecanol (TD). The composite PCMs could be triggered by temperature variations with good reversible thermochromic ability due to the introduction of TC compounds. When the temperature was increased from 25 to 50 °C, the color of the thermochromic composite PCMs evolved from dark blue to light blue and then to off‐white. Li et al.^[^ ^158^ ^]^ fabricated lightweight and highly electrically conductive 3D CAs from fresh winter melons through a hydrothermal carbonization process (Figure 27d). The latent heat of the corresponding composite PCMs was 115.2 J g^−1^. Moreover, the winter melon derived carbon‐based composite PCMs could be used for electric‐to‐thermal and solar‐to‐thermal conversion (Figure 27e). As a result, the electric‐to‐thermal conversion efficiency was 71.4% at a low voltage due to highly electrically conductive CAs (6.5 S m^−1^) and the solar‐to‐thermal conversion efficiency was 96% due to high solar adsorption over the whole UV–vis–NIR range.

### 5.6 Expanded Graphite‐Based Composite PCMs

EG has been extensively researched for the encapsulation of organic PCMs due to its large specific surface area, strong adsorption capability, and high thermal conductivity.^[^ ^173^, ^174^, ^175^ ^]^ In the composite PCMs, EG can not only serve as a supporting material to solve the leakage of PCMs due to its strong adsorption capacity and intercalation effect, but also improve the thermal conductivity of PCMs due to its continuous carbon network structure. The thermal stability of composite PCMs can also be greatly improved by combining EG and flame retardant materials.^[^ ^176^ ^]^ In addition, the synergistic flame retardant effect of EG and flame retardant materials can be utilized for the preparation of fire‐proof materials for the thermal management in building applications.^[^ ^177^, ^178^ ^]^

**Figure 28:**
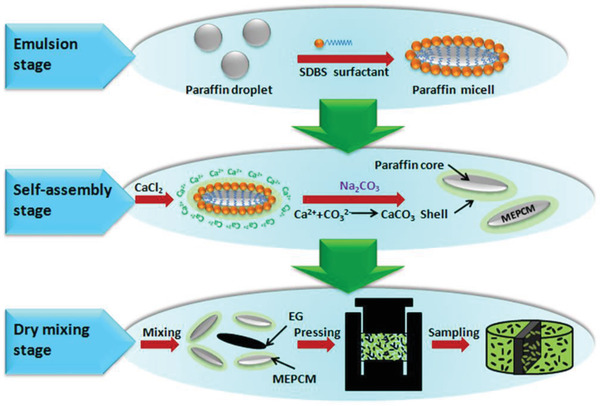
Preparation diagram of the microencapsulated PCMs. Reproduced with permission.^[^ ^173^ ^]^ Copyright 2017, Elsevier.

With respect to TES, thermal conductivity is a crucial evaluation factor for assessing the heat storage/release rate and energy storage efficiency. Li et al.^[^ ^179^ ^]^ introduced EG into SA to obtain a high‐performance tankless solar water heater using a melting impregnation method. The adsorption capacity of SA reached 98 wt% with an energy storage density of 163.5 J g^−1^. The thermal conductivity of composite PCMs loaded with 6 wt% EG dramatically increased to 2.50 W mK^−1^, which was ≈9.6 times higher than that of pure SA. The heat release time of the EG‐modified composite PCMs was 74.8% less than that of pure SA. Wang et al.^[^ ^173^ ^]^ also investigated the thermal conductivity of MEPCMs composed of paraffin and CaCO_3_ through a precipitation reaction of CaCl_2_ and Na_2_CO_3_, followed by the introduction of EG using pressing machine (**Figure** **28**). As a result, the thermal conductivity of MEPCMs with 24 wt% EG reached 8.86 W mK^−1^ due to the constructed dense and continuous carbon network structure,^[^ ^85^, ^180^ ^]^ which was ≈24 times higher than that of pure paraffin. Yin et al.^[^ ^181^ ^]^ constructed 3D‐graphite blocks using a self‐assembly method and then prepared graphite block based composite PCMs using a casting method. The obtained composite PCMs exhibited a high thermal conductivity of 15.6 W mK^−1^ and a low volume expansion of <1% due to the continuous 3D‐graphite network.

As previously mentioned, MOF derivatives have an excellent 3D interpenetrating network structure, which can provide effective hierarchical thermal transfer paths for PCMs. Based on this advantage of MOF derivatives, Li et al.^[^ ^53^ ^]^ constructed composite PCMs using EG and hollow porous Co_3_O_4_ derived from ZIF‐67 via the calcination of ZIF‐67 and in‐situ deposition on EG. After the introduction of SA into the 3D porous interconnected network structure, the resulting composite PCMs exhibited a high latent heat of 218.6 J g^−1^ and a faster thermal response than pure SA due to the effective hierarchical thermal transfer paths from Co_3_O_4_/EG.^[^ ^182^ ^]^ Moreover, the thermal conductivity of composite PCMs loaded with 10 wt% Co_3_O_4_/EG reached 2.53 W mK^−1^, which corresponds to an increase of 767%.

**Figure 29:**
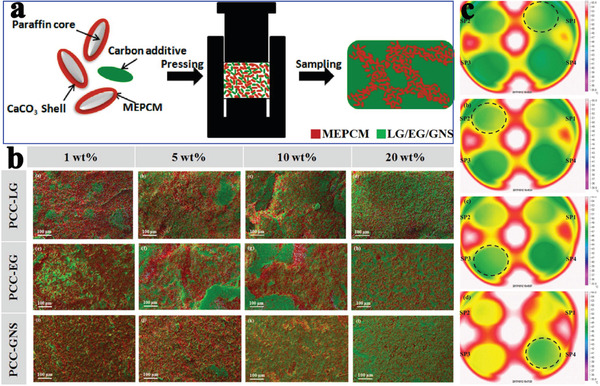
a) Preparation diagram of the microencapsulated PCMs. b) EDS mappings of the microencapsulated PCMs with different mass fractions of carbon. c) Thermal infrared images. Reproduced with permission.^[^ ^174^ ^]^ Copyright 2018, Elsevier.

To further study the heat transfer enhancement mechanism of EG, Wang et al.^[^ ^174^ ^]^ introduced different mass fractions of EG into MEPCMs (**Figure** **29**a). The attachment structure of carbon additives increases with increasing EG concentration. As shown in Figure 29b, a distinct thermal conduction network was formed by the connection of the conductive chains. It is worth noting that EG was difficult to disperse uniformly when the content was less than 20 wt%, which is due to its larger particle size and length than those of microcapsules. Microcapsules (1–8 µm) were appropriate for filling the internal pores of EG, thereby reducing the contact thermal resistance caused by air filling. As a result, the thermal conductivity of MEPCMs reached 25.81 W mK^−1^, which was 70 times that of pure paraffin. Thermal images (Figure 29c) further indicated that constructing 3D network structure was essential to accelerating heat transfer in different directions. Additionally, the change in the thermal performance of MPCMs was negligible after 500 thermal cycling tests due to the extraordinarily stable 3D network structure.^[^ ^23^, ^85^ ^]^

**Figure 30:**
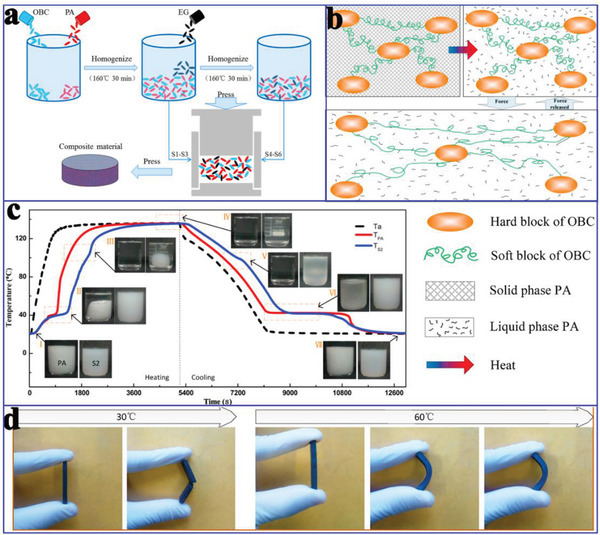
a) Preparation diagram of composite PCMs. b) Schematic diagram of the deformation. c) Temperature‐time curves of composite PCMs. d) Schematic diagram of the bending deformation of composite PCMs. Reproduced with permission.^[^ ^184^ ^]^ Copyright 2019, Elsevier.

However, strong rigidity usually emerges in the single EG‐based composite PCMs. To solve the rigidity problem, EG and other supporting materials should be applied together.^[^ ^175^ ^]^ Li et al.^[^ ^183^ ^]^ encapsulated paraffin into a difunctional olefin block copolymer (OBC) with a physically crosslinked network and macroscopic elasticity. In addition, EG was embedded to enhance the thermal conductivity of the flexible composite PCMs.^[^ ^85^ ^]^ Surprisingly, the thermal conductivity of composite PCMs with only 3 wt% EG loading reached 1.68 W mK^−1^ with a breakthrough increase of 479%. Wu et al.^[^ ^184^ ^]^ reported thermally induced flexible PCMs composed of OBC, EG, and paraffin (**Figure** **30**a,b). The porous EG not only provided support functionality through capillary force and surface tension^[^ ^184^, ^185^ ^]^ and but also was also chosen as the thermal conductivity enhancer. The resulting composite PCMs exhibited a high TES density of 197.7 J g^−1^ owing to the high encapsulation efficiency of paraffin (79.2 wt%). The thermal conductivity of paraffin/OBC/EG composite PCMs was 5.50 W mK^−1^ when the mass fraction of EG was 10% (Figure 30c). More importantly, the composite PCMs could be transformed into flexible blocks and exhibited various deformation modes (Figure 30d) when the temperature was above the melting point of PA because the liquid phase could cause a large variation in the chain mobility of the continuous phase.^[^ ^182^, ^186^ ^]^ Lin et al.^[^ ^175^ ^]^ used polyvinyl butyral (PVB) to prepare palmitic acid‐based composite PCMs with enhanced thermal conductivity due to the blending EG. The latent heat of composite PCMs was 128.08 J g^−1^ and the thermal conductivity was improved by 4.2 times due to the addition of 7 wt% EG.

**Figure 31:**
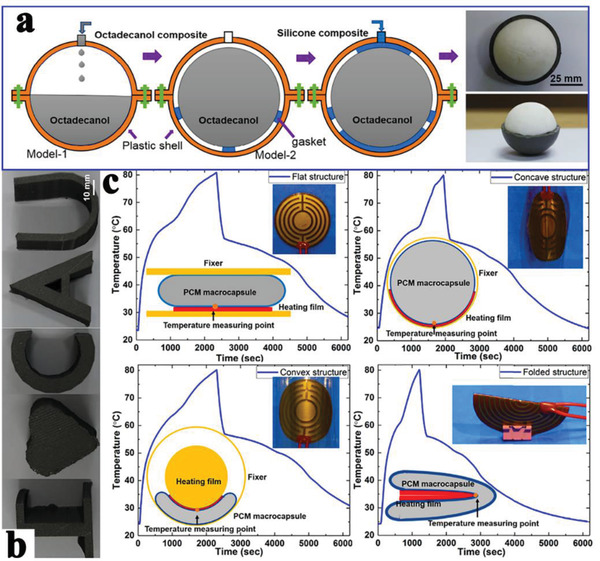
a) Preparation diagram of the phase change macrocapsules. b) Different shapes of macrocapsules. c) Temperature‐time curves of phase change macrocapsules‐based heat sink with different shapes of flat, concave, convex, and folded structures. Reproduced with permission.^[^ ^187^ ^]^ Copyright 2019, Elsevier.

In addition to the traditional gel–gel method for preparing microscopic phase change microcapsules, Yu et al.^[^ ^187^ ^]^ reported macroscopic phase change microcapsules composed of a silicone elastomer shell and an octadecanol core using a cast molding method (**Figure** **31**a). The supporting materials could not only can effectively encapsulate octadecanol, but also repeatedly reshape into complex shapes with large‐scale deformation as needed (Figure 31b). The thermal conductivity of the macrocapsules loaded with 2 wt% EG was 1.53 W mK^−1^, which was a nearly 4.28‐fold increase in comparison with that of pure octadecanol. The thermal conductivity of macrocapsules loaded with 1 wt% EG and Bi–In–Sn eutectic alloys was 1.98 W mK^−1^, which corresponded to an 890% increase over that of pure silicone. Moreover, the prepared core–shell macrocapsules exhibited a high latent heat density of 210.1 MJ m^−3^ and excellent self‐adaptative deformation ability. Additionally, the PCMs‐based macrocapsules with different shapes (Figure 31c) could be used for the thermal management of the complicated electronic devices.

In addition to the aforementioned thermal storage and heat transfer performance studies of EG‐based composite PCMs, multifunctional composite PCMs are destined to be more popular for future applications. Integrating different functional materials is a feasible strategy. For example, the perfect synergistic effect of EG and fire‐retardant materials can be utilized to prepare fire‐proof materials for the thermal management in building fields.^[^ ^176^, ^177^, ^178^, ^185^ ^]^ In a study by Fang et al.,^[^ ^188^ ^]^ SiO_2_ was used to fabricate *n*‐hexadecane‐based composite PCMs with enhanced flame retardant properties. This behavior was achieved because the surface carbonaceous‐silicate charred layer could insulate the underlying materials and retard the escape of the volatile products formed during the thermal degradation.^[^ ^177^, ^178^, ^189^ ^]^ Moreover, the composite PCMs exhibited high melting and solidifying latent heats of 147.58 and 145.10 J g^−1^ respectively. Similarly, Cai et al.^[^ ^176^ ^]^ prepared a flame retardant system with paraffin/high‐density polyethylene (HDPE) composites using a twin‐screw extruder technique based on the synergistic effect of EG and ammonium polyphosphate. In addition, Tabassum et al.^[^ ^190^ ^]^ reported electric‐to‐thermal energy conversion PCMs combining EG and methyl stearate. As a result, the electric‐to‐thermal energy conversion PCMs with a conversion efficiency of 72% were triggered at a low voltage of 1.4 V.

In addition to EG‐based organic composite PCMs, Yuan et al.^[^ ^191^ ^]^ prepared EG/Ba(OH)_2_·8H_2_O inorganic composite PCM. The EG was modified with octylphenol polyoxyethylene to improve its compatibility with Ba(OH)_2_·8H_2_O. As a result, EG not only reduced the supercooling degree from 13 to 2.4 °C, but also effectively inhibited the phase separation. Moreover, EG contributed substantially to the high thermal conductivity (3.58 W mK^−1^) of composite PCM, which was 184% higher than that of Ba(OH)_2_·8H_2_O. Zhang et al.^[^ ^192^ ^]^ prepared polymer‐coated CaCl_2_·6H_2_O/EG composite PCMs with superior thermal stability and thermal reliability. Furthermore, Yuan et al.^[^ ^193^ ^]^ prepared solar‐to‐thermal conversion composite PCM containing EG, CH_3_COONa·3H_2_O, Na_2_HPO_4_, and CuS. Na_2_HPO_4_ served as an effective nucleating agent that greatly promoted the crystallization of CH_3_COONa·3H_2_O. CuS further improved the solar‐to‐thermal conversion efficiency from 66.9% to 94.1%. Moreover. the resultant composite PCMs showed high latent heat of 194.8 J g^−1^, low supercooling degree and long service life.

In addition to single EG‐based inorganic composite PCMs, Wang et al.^[^ ^194^ ^]^ synthesized three different EG/eutectic binary molten salts (LiNO_3_‐KCl, LiNO_3_‐NaNO_3_, and LiNO_3_‐NaCl) composite PCMs. After EG was impregnated, the thermal conductivity of the eutectic binary molten salts increased by up to 4.9–6.9 times. Yang et al.^[^ ^195^ ^]^ impregnated Na_2_CO_3_·10H_2_O‐Na_2_HPO_4_·12H_2_O eutectic hydrated salts into EG and expanded graphite oxide (EGO). Compared with EG‐based composite PCMs, EGO‐based composite PCMs exhibited a higher latent heat, lower supercooling degree, and better thermal stability. More importantly, to reach the same level of thermal conductivity as that of composite PCMs, the content of EG was approximately twice as high as the content of EGO.

### 5.7 Other Carbon‐Based Composite PCMs

**Figure 32:**
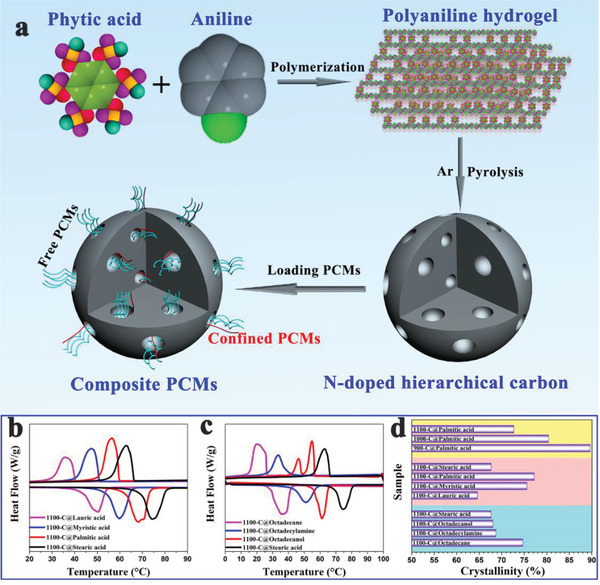
a) Preparation diagram of N‐doped hierarchical porous carbon‐based composite PCMs. b,c) DSC curves of different composite PCMs. d) Crystallinities of different composite PCMs. Reproduced with permission.^[^ ^134^ ^]^ Copyright 2019, Elsevier.

The nanoconfinement behaviors of organic PCMs in the nanoscale PC materials greatly affect the thermal energy utilization efficiency when undergoing a phase change. Chen et al.^[^ ^134^ ^]^ comprehensively researched the influences of interfacial interactions between N‐doped hierarchical carbon and different small molecular organic PCMs on the thermal storage performance. In situ N‐doped 3D nanoscale HPC was prepared via one‐step high temperature pyrolysis of polyaniline hydrogel. The encapsulation process of the N‐doped carbon‐based composite PCMs was shown in **Figure** **32**a. The thermal conductivity of composite PCMs gradually increased with increasing pyrolysis temperature, which was attributed to the synergistic effect of highly graphitic nitrogen and interconnected graphitized network carbon.^[^ ^196^, ^197^, ^198^ ^]^ These findings verified that one‐step pyrolysis derived in situ N‐doped hierarchical network carbon was beneficial for accelerating the phonon transport. Differential scanning calorimetry (DSC) results (Figure 32b–d) indicated that the interactions at the interfaces between organic small molecular PCMs and N‐doped carbon determined the nanoconfinement behaviors, which mainly relied on the hydrogen bond intensity and space restriction effect.^[^ ^134^, ^199^, ^200^ ^]^ This mechanism offered leading insights into the direct preparation of high‐performance organic composite PCMs. However, this mechanism is not known for the use of inorganic PCMs for TES and utilization.

**Figure 33:**
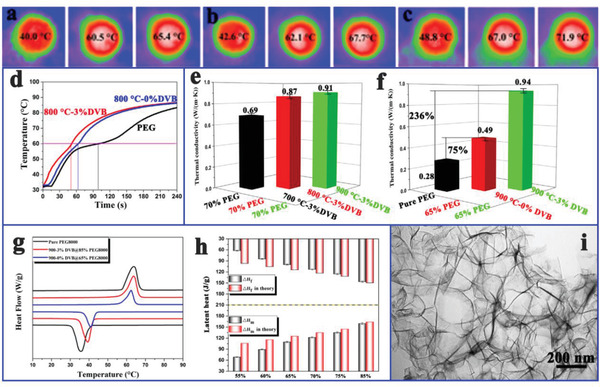
a–c) Thermal infrared images of various composite PCMs. d) Temperature–time curves of pure PEG and composite PCMs. e,f) Thermal conductivities of pure PEG and composite PCMs. g) DSC curves of pure PEG and composite PCMs. h) Latent heat values of various composite PCMs. i) TEM image of CQDs‐derived porous carbon. Reproduced with permission.^[^ ^24^ ^]^ Copyright 2018, Elsevier.

Highly thermally conductive additives are usually introduced into a system to enhance the thermal conductivity of composite PCMs. However, inadequate additives achieve only limited thermal conductivity enhancement effects owing to the large interfacial thermal resistance. Conversely, although adequate additives can significantly improve the thermal conductivity, the thermal storage density is considerably reduced due to the occupation of additives inside original channels.^[^ ^201^, ^202^, ^203^, ^204^ ^]^ Therefore, excellent thermal storage and thermal conductivity are difficult to achieve. Based on this challenge, our group constructed a 3D compactly interconnected highly graphitized network carbon via one‐step calcination of CQDs.^[^ ^24^ ^]^ The pore structure was effectively regulated under the synergistic effect of the crosslinking reaction and pyrolysis temperature. Importantly, PEG could be uniformly infiltrated into the 3D highly graphitized network carbon and fully stretched and crystallized. Hence, the resulting composite PCMs simultaneously integrated superior thermal storage and thermal conductivity capacity (**Figure** **33**a–h). The latent heats approached the theoretical values. The thermal conductivity of the composite PCMs was 236% higher than that of pure PEG, which was attributed to the substantial phonon propagation vibration of the highly graphitized interconnected network carbon (Figure 33i).^[^ ^57^, ^205^, ^206^ ^]^ In addition, this strategy could obviously reduce the sacrifice degree of thermal storage density, compared with that associated with directly introducing large amounts of highly thermally conductive additives to improve the thermal conductivity.

**Figure 34:**
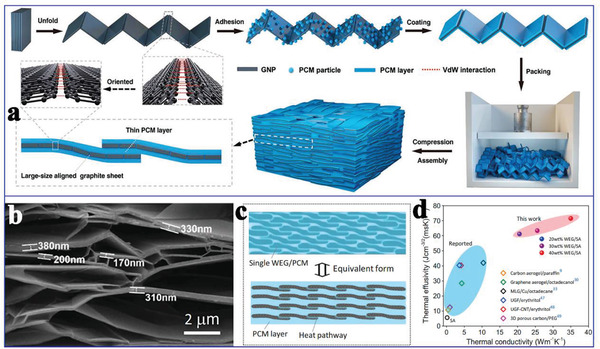
a) Preparation diagram of composite PCMs and large‐size aligned graphite sheets. b) SEM image of 3D aligned and interconnected graphite nanoplatelets framework. c) Schematic diagram of the parallel model for the thermal conduction. d) Thermal effusivity comparison with the latest reports. Reproduced with permission.^[^ ^207^ ^]^ Copyright 2019, Wiley‐VCH.

As previously stated, EG has great potential in improving the thermal conductivity of PCMs. Aligned graphite sheets can be obtained by mechanically compressing EG. In a study by Wang et al.,^[^ ^207^ ^]^ large‐size aligned graphite sheets from worm‐like EG were constructed inside PCMs using a compression‐induced method (**Figure** **34**a). The resulting graphite sheets at the millimeter scale were composed of highly oriented van der Waals‐bonded graphite nanoplatelets at the micrometer scale (Figure 34b). Van der Waals interactions among adjacent graphite nanoplatelets can weaken the interfacial phonon scattering due to the small adhesion energy (<100 mJ m^−2^), thereby reducing the interfacial thermal resistance and improving the thermal conductivity.^[^ ^208^ ^]^ As a result, the thin PCM layers between adjacent graphite sheets exhibited an ultrahigh thermal conductivity ranging from 4.4 to 35.0 W mK^−1^ with less than 40.0 wt% graphite due to the synergistic contribution of low junction thermal resistance and low spatial density (Figure 34c,d). This strategy provided a promising route to design high‐power‐density and low‐cost composite PCMs in the large‐scale thermal storage and management of electronics.

**Figure 35:**
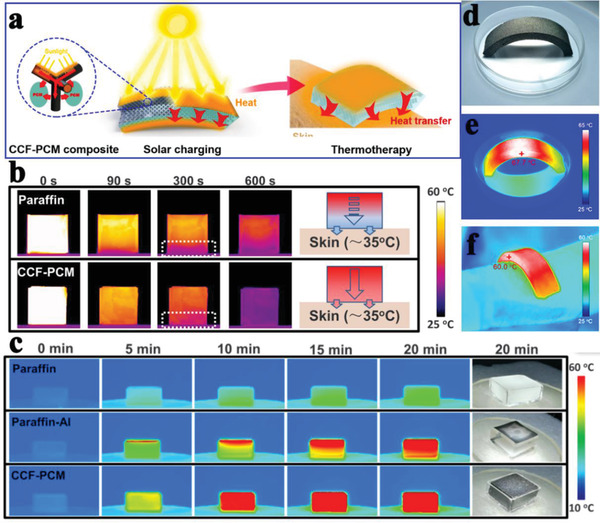
a) Schematic diagram of composite PCMs for thermal comfort application. b) IR images of pure paraffin and CCF‐paraffin composite PCMs. c) IR images of pure paraffin, paraffin‐Al, and CCF‐paraffin composite PCMs. d) Photograph of a bent CCF‐paraffin composite heat pack. e,f) IR images of CCF‐paraffin composite heat pack on the human arm. Reproduced with permission.^[^ ^209^ ^]^ Copyright 2018, American Chemical Society.

To date, the single thermal function of composite PCMs has difficulty in meeting specific functional requirements in some particular devices. To develop advanced multifunctional composite PCMs, Ye et al.^[^ ^209^ ^]^ designed a heat pack for human body comfort and thermotherapy by impregnating paraffin into carbon‐coated copper foams (CCFs) (**Figure** **35**a). Black nanoparticles and uncured polydimethylsiloxane (PDMS) were coated onto the oxidized copper foam. The supporting materials smartly integrated robust PDMS coatings and solar absorbing carbon nanoparticles after curing. The hydrophobic surface of CCFs effectively confined the liquid paraffin without leakage. Simulated human skin results revealed that the CCFs‐paraffin composite PCMs took 313 s to cool down to the healing temperature of 40 °C, more than 18 times longer than pure paraffin, thereby prolonging the thermotherapy time. Even though more thermal energy was stored in the pure paraffin (≈975 J) than in the CCFs‐paraffin composite PCMs (≈925 J) with the same size, the extractable heat from CCFs‐paraffin composite PCMs was significantly higher than that of pure paraffin. Similarly, the charging performance of CCFs‐paraffin composite PCMs was investigated (Figure 35b,c). The results clearly showed that the temperature difference at the top surface, center, and bottom of CCFs‐paraffin composite PCMs was negligible during the charging process. In addition, CCFs‐paraffin composite PCMs could be bent into different sizes to fit the curvature of the human wrist and release heat energy evenly to the skin once charged (Figure 35d–f). These results confirmed the thermal comfort application of CCFs‐paraffin composite PCMs on the human wrist.

**Figure 36:**
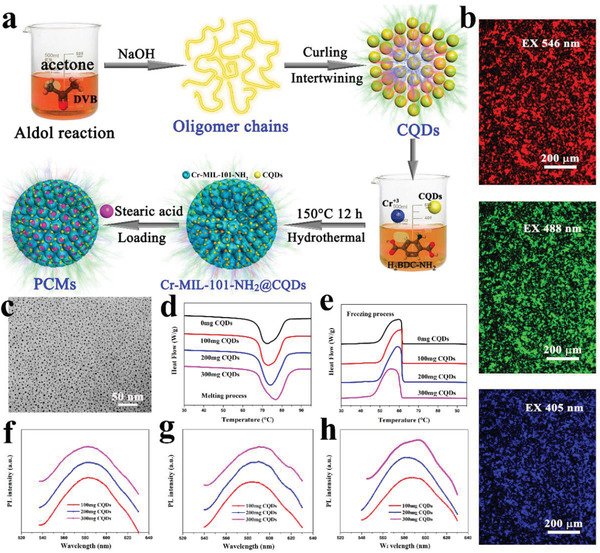
a) Preparation diagram of PL‐functionalized composite PCMs. b) Fluorescent images of CQDs. c) HRTEM image of CQDs. d,e) DSC curves of MOF‐based composite PCMs. f–h) PL spectra of MOF‐based composite PCMs. Reproduced with permission.^[^ ^128^ ^]^ Copyright 2019, Elsevier.

Our group designed photoluminescence (PL)‐functionalized MOF‐based composite PCMs by incorporating CQDs (≈2.7 nm) into Cr‐MIL‐101‐NH_2_ (**Figure** **36**a–c).^[^ ^128^ ^]^ To the best of our knowledge, this is the first time advanced multifunctional MOF‐based fluorescent composite PCMs was developed. Fluorescent guest molecules are mainly ionic type, and aggregation‐induced quenching of organic phosphors often emerges in their solid states.^[^ ^210^ ^]^ This uniquely designed structure not only inhibited internal molecular motion to impede nonradioactive relaxation but also avoided aggregation‐induced fluorescent quenching.^[^ ^211^, ^212^ ^]^ MOF framework can efficiently transfer energy to the embedded CQDs.^[^ ^213^ ^]^ The resulting MOF‐based composite PCMs not only exhibited excellent thermal storage performance but also emitted outstanding red–green–blue multicolor fluorescence (Figure 36d,e). This fluorescence phenomenon is closely associated with surface energy traps, surface functional groups and electron–hole recombination induced by the quantum size effect.^[^ ^214^, ^215^, ^216^ ^]^ Moreover, fluorescence still existed whether PEG, octadecanol or SA were infiltrated into Cr‐MIL‐101‐NH_2_/CQDs (Figure 36f–h). This host–guest strategy provided a novel platform for developing advanced multifunctional MOF‐based composite PCMs. However, the development of MOF‐based multifunctional composite PCMs is still in its infancy and there are many unimaginable challenges. The mutual matching relationship of MOF hosts and functional guests should be explored in detail.

## 6 Conclusions and Prospects

The extensive utilization of TES technologies based on PCMs, especially high‐performance carbon‐based composite PCMs, can effectively promote the development of renewable and sustainable energy. However, composite PCMs still face some challenges, such as relatively low TES density, low thermal conductivity, and low energy conversion efficiencies, especially electric‐to‐thermal conversion and magnetic‐to‐thermal conversion efficiencies. In addition, advanced multifunctional composite PCMs must be further developed to meet the needs of specific scenarios. To provide constructive insights into these issues, herein, we systematically summarize the advances in composite PCMs based on different carbon materials (CNTs, CFs, graphene/GO/rGO, MOF‐derived carbon, biomass‐derived carbon, EG, and other forms of carbons) for TES, thermal transfer, energy conversion, and advanced utilization. The corresponding engineering regulation strategies and microscopic mechanisms regarding the thermal performances of carbon‐based composite PCMs had been analyzed. The TES capability of carbon‐based composite PCMs mainly depends on the size and structure of the carbon materials and the interactions between the carbon materials and PCMs. The thermal conductivity of carbon‐based composite PCMs mainly depends on the graphitization degree and structural regularity of the carbon materials and the types and loading contents of PCMs. The solar‐to‐thermal, electric‐to‐thermal, and magnetic‐to‐thermal energy conversion efficiencies of carbon‐based composite PCMs mainly depend on the solar absorption capacity and electronic conductivity of carbon materials and the magnetic response capability of the doped magnetic nanoparticles.

In terms of the TES capability of carbon‐based composite PCMs, EG is the most promising candidate. In terms of the thermal transfer capability of carbon‐based composite PCMs, array‐oriented graphene and CNTs network are the most promising candidates. In terms of the energy conversion efficiency of carbon‐based composite PCMs, solar‐to‐thermal conversion is currently relatively mature, and the conversion efficiency has reached a very high level. Comparatively, the electric‐to‐thermal conversion efficiency of carbon‐based composite PCMs is relatively low. The realization of magnetic‐to‐thermal conversion requires doped magnetic nanoparticles. However, very few studies have been performed on magnetic‐to‐thermal conversion, which is still in its infancy. Additionally, some other advanced multifunctional applications of carbon‐based composite PCMs had been mentioned, such as controlled drug release, fluorescence, and thermotherapy functions. Despite the recent significant advancements in carbon‐based composite PCMs for TES, thermal transfer, and energy conversion, several issues still require further research and resolution in the further, mainly including the following key points.

1) Current strategy for improving the TES capacity of carbon‐based composite PCMs mainly focuses on regulating the pore size of carbon materials. The corresponding studies are relatively few concerning the functional groups‐modified pores, pore shapes, and hierarchical pore distributions of carbon materials, and require further research.
2) Compared to disordered carbon materials, array‐oriented carbon materials are more conducive to effectively integrating high TES and heat transfer capabilities. Therefore, simple and easy‐to‐use synthesis strategies for array‐oriented carbon materials are worth further exploration.
3) Although many experimental results on the thermal conductivity enhancement of PCMs using carbon materials are reported, most are still deficient in the in‐depth mechanism exploration. Insights into the mechanisms are conducive to guiding the targeted preparation of carbon‐based composite PCMs with high thermal conductivity.
4) Compared to high‐efficiency solar‐to‐thermal energy conversion, electric‐to‐thermal energy conversion efficiency of carbon‐based composite PCMs is relatively low. Therefore, it is an indispensable task to further improve the electric‐to‐thermal energy conversion efficiency.
5) Doped magnetic nanoparticles in carbon‐based composite PCMs can realize the magnetic‐to‐thermal energy conversion; however, this design technology is new, and the corresponding researches are still few. In addition, current magnetic‐to‐thermal energy conversion efficiency is still very low. Therefore, developing high‐efficiency magnetic‐to‐thermal energy conversion composite PCMs is urgent.
6) More optimized calculation methods for solar‐to‐thermal, electric‐to‐thermal, and magnetic‐to‐thermal energy conversion efficiencies need to be further explored, because current calculation methods do not consider the heat loss of the test setups in the exposed external environment.
7) To satisfy specific application requirements, advanced multifunctional carbon‐based composite PCMs should be developed and optimized, such as mechanical, optical, electrical, magnetic, acoustic, biological, and chemical functionalities.

## Conflict of Interest

The authors declare no conflict of interest.
